# Genetic Assessment of Neurotoxicity Accompanied by Inhalational Anesthesia in Preclinical Studies with Focus on Sevoflurane and Isoflurane—A Narrative Review

**DOI:** 10.3390/brainsci16070661

**Published:** 2026-06-23

**Authors:** Milena Djordjevic, Jovan Milosavljevic, Marina Mitrovic, Miodrag Sreckovic, Dragica Selakovic, Ana Maksimovic Sreckovic, Gvozden Rosic

**Affiliations:** 1 Department of Physiology, Faculty of Medical Sciences, University of Kragujevac, 34000 Kragujevac, Serbia; djordjevic.milena.90@gmail.com (M.D.); jovan.milosavljevic1997@gmail.com (J.M.); grosic@fmn.kg.ac.rs (G.R.); 2 Department of Medical Biochemistry, Faculty of Medical Sciences, University of Kragujevac, 34000 Kragujevac, Serbia; mitrovicmarina34@gmail.com; 3 Center for Molecular Medicine and Stem Cell Research, Faculty of Medical Sciences, University of Kragujevac, 34000 Kragujevac, Serbia; 4 Department of Internal Medicine, Faculty of Medical Sciences, University of Kragujevac, 34000 Kragujevac, Serbia; sreckovic7@gmail.com; 5 Clinic of Cardiology, University Clinical Center Kragujevac, 34000 Kragujevac, Serbia; 6 Department of Anesthesiology and Resuscitation, University Clinical Center Kragujevac, 34000 Kragujevac, Serbia; anamsreckovic@gmail.com

**Keywords:** inhalational anesthesia, neurotoxicity, sevoflurane, isoflurane, gene alterations, preclinical investigation

## Abstract

**Highlights:**

**What are the main findings?**

- The identification of genes whose altered expression contributes to anesthetic-induced neurotoxicity.
- Preclinical studies have shown the age-related differences in anesthesia-induced neurotoxicity susceptibility, especially during neurological development and in aging animal models.

**What are the implications of the main findings?**

- The identification of specific gene expression changes and molecular pathways involved in anesthetic neurotoxicity could enable the development of neuroprotective strategies and the discovery of biomarkers for risk assessment.
- Evidence from animal studies supports consideration of the dose, timing, and duration of inhalational anesthesia, especially in neonatal, pregnant, and aged animal models.

**Abstract:**

Inhalational anesthesia, which includes anesthetics such as sevoflurane, isoflurane, and desflurane, is widely used in clinical settings for surgical interventions across all age groups. Nonetheless, recent findings from preclinical research raise important questions regarding their potential neurotoxic effects, especially within the developing brain, though clinical implications remain to be fully established. This narrative review was conducted through a literature search of the PubMed database and synthesizes preclinical investigations into gene modifications associated with neurotoxicity following exposure to inhalation anesthetics. Emphasis was placed on anesthetic exposure in human and animal-derived cell lines, neurodevelopmental animal models, as well as adult and aged animals. In various models, the neurotoxic mechanisms of inhalational anesthesia involve a complex interaction of apoptosis, oxidative stress, mitochondrial dysfunction, neuroinflammation, and epigenetic remodeling. Developmental studies indicate additional susceptibilities, including impaired neuronal migration, myelination deficits, and transgenerational epigenetic effects, whereas aging models exhibit oxidative stress injury, microglial activation, and heightened perioperative neurocognitive sensitivity. Understanding these neurotoxic mechanisms is essential for identifying risk factors, formulating age-specific neuroprotective strategies, and enhancing the overall safety of anesthetic use, particularly in vulnerable populations.

## 1. Introduction

Anesthesia is necessary for nearly all surgical and diagnostic procedures. Each year, approximately 230 million anesthetic procedures are performed worldwide [1]. In the United States alone, over 50 million surgical procedures are conducted annually, with anesthesia playing a crucial role in perioperative care [2]. According to a broadly accepted classification, anesthesia is defined as general, regional, local, and sedation types [3].

General anesthesia represents a pharmacologically induced reversible state characterized by unconsciousness, analgesia, amnesia, and muscle relaxation, thereby enabling the performance of invasive surgical procedures under controlled physiological conditions. It is achieved through the administration of intravenous and/or inhalational agents that primarily modulate neuronal signaling through γ-aminobutyric acid (GABA), N-methyl-D-aspartate (NMDA), glycine, and ion channel-associated signaling pathways [4]. General anesthesia can be classified by the route of administration and anesthetic maintenance strategy as intravenous, inhalational, balanced, and total intravenous anesthesia (TIVA). Intravenous anesthesia involves the administration of anesthetic agents (propofol, ketamine, etomidate, benzodiazepines, and barbiturates) directly into the systemic circulation to induce rapid loss of consciousness [5,6]. Inhalational anesthesia is achieved by administering volatile anesthetic agents or gases via inhalation. Frequently used inhalational anesthetics are sevoflurane, isoflurane, desflurane, and nitrous oxide [7]. These agents allow continuous adjustment of anesthetic depth because their concentrations can be titrated in accordance with the patient’s physiological status and surgical requirements. Balanced anesthesia is the most commonly used anesthetic approach in clinical practice and involves induction with intravenous agents, followed by application of inhalational anesthetics, opioids, neuromuscular blocking agents, and adjuvant medications to achieve general anesthesia while reducing the adverse effects of individual drugs [8]. TIVA refers to the maintenance of general anesthesia exclusively through the continuous intravenous administration of anesthetic agents, without the use of inhalational anesthetics [9,10]. Regional anesthesia is characterized by a reversible loss of sensory and, in some cases, motor function within a larger anatomical region, achieved through the targeted blockade of peripheral nerves, nerve plexuses, or spinal nerve roots [11,12]. Compared with general anesthesia, it preserves consciousness and has fewer side effects [13]. Local anesthesia induces a transient loss of sensation in a restricted area of the body without affecting the patient’s consciousness or CNS functions and is widely used in minor surgical, dental, dermatological, and ophthalmological procedures [14,15,16]. Procedural sedation represents the use of sedative, anxiolytic, and analgesic medications to facilitate diagnostic or therapeutic procedures that a fully conscious person would find intolerable. Depending on the depth, procedural sedation may range from minimal anxiolysis to deep sedation approaching general anesthesia [17,18].

## 2. Inhalational Anesthetics

The aim of this narrative review was to analyze the impact of inhalational anesthetics on gene alterations that may be accompanied by neurotoxicity. Inhalational anesthetics are volatile agents or gases delivered by inhalation to induce and maintain anesthesia (typically included in balanced anesthesia protocols), and they are essential in perioperative medicine due to their adjustability, controllable depth, and predictable removal via respiration [19]. In modern medical practice, the most widely used inhalational anesthetics are sevoflurane, isoflurane, desflurane, and nitrous oxide [8]. Halothane was discontinued because of serious side effects [20], and enflurane is rarely used now, primarily due to cardiovascular risks and its effects on seizure threshold [21]. These drugs act through complex interactions within targets in our CNS, including GABAA, glycine, and NMDA receptors, as well as potassium and other ion channels [4]. By modulating neuronal activity, they cause reversible unconsciousness, amnesia, pain relief, and immobility, while also dampening autonomic responses. Their pharmacokinetics depend on blood-gas partition coefficients, which affect the rate at which anesthesia takes effect and wears off; agents with lower blood solubility act faster, leading to quicker induction and recovery [22]. Beyond their anesthetic effects, these agents can also influence cerebral blood flow, heart function, breathing, and brain metabolism [7]. Additionally, because they affect neuronal networks and signaling pathways, researchers are studying their broader impacts on neurodevelopment, inflammation, and cell survival [23].

Nitrous oxide is a non-halogenated inhalational anesthetic gas with potent analgesic properties, yet it has relatively weak anesthetic potency when used alone [24]. The anesthetic effect of nitrous oxide results from its antagonism of NMDA receptors and the modulation of endogenous opioid pathways [24]. Due to its rapid onset and low blood solubility, nitrous oxide is commonly used as an adjunctive anesthetic agent, in combination with either volatile or intravenous anesthetics, to reduce anesthetic requirements and enhance perioperative analgesia [25].

Sevoflurane is currently among the most frequently used inhalational anesthetics because of its low blood-gas solubility, rapid induction and recovery, minimal airway irritation, and relatively favorable cardiovascular stability [26]. Due to its pleasant odor and smooth inhalational induction characteristics, sevoflurane is especially suitable for pediatric anesthesia and ambulatory surgical procedures [27]. A limited amount of administered sevoflurane is metabolized in the liver, predominantly by cytochrome P450 enzymes, resulting in the production of inorganic fluoride ions and compound A as metabolites [28]. Because of its widespread use, sevoflurane has become one of the most extensively studied inhalational anesthetics in preclinical neurotoxicity research.

Isoflurane is a potent halogenated volatile anesthetic that is widely used for maintenance of general anesthesia due to its reliable anesthetic efficacy and relative hemodynamic stability. Compared with sevoflurane, isoflurane is associated with greater airway irritability and a slower induction profile due to its higher blood-gas partition coefficient [22]. In clinical practice, isoflurane remains useful in prolonged surgical procedures because of its cardiovascular stability and relatively low metabolic degradation [29].

Desflurane has an exceptionally low blood-gas partition coefficient, resulting in extremely rapid induction and emergence from anesthesia [30], making it highly suitable for outpatient procedures and surgeries requiring rapid postoperative neurological assessment [31]. However, desflurane may cause airway irritation, coughing, laryngospasm, and sympathetic stimulation when administered at high concentrations or during inhalational anesthesia induction [32].

## 3. Adverse Effects of Inhalation Anesthesia

Inhalational anesthetics remain essential in perioperative medicine, but they have been linked to a broad range of adverse systemic and neurological effects. Volatile anesthetics (isoflurane, sevoflurane, and desflurane) are widely regarded as safe and effective; however, accumulating preclinical and clinical evidence suggests the possibility of both acute and long-term complications, particularly in animal models of the young and elderly. Whether these findings translate directly to human patients, especially those with pre-existing neurological impairment, remains an area of active investigation. Some of the side effects include postoperative nausea and vomiting, cardiovascular depression, hypotension, impaired respiratory function, and changes in cerebral blood flow regulation. Recent research has focused on their potential neurotoxic properties and their involvement in postoperative cognitive dysfunction (POCD) and delirium [33]. Recent experiments have shown that inhalational anesthetics can induce neuronal apoptosis, mitochondrial dysfunction, oxidative stress, calcium dysregulation, and excessive ROS production, thereby impairing neuronal survival and synaptic integrity. Additionally, inhalational anesthetics have been shown to activate microglia and promote neuroinflammatory responses, characterized by an increased expression of pro-inflammatory cytokines, including interleukin-1β (IL-1β), interleukin-6 (IL-6), and tumor necrosis factor-α (TNF-α), thereby exacerbating neuronal injury and cognitive deficits [34,35]. Several studies have demonstrated that inhalational anesthetics accelerate processes associated with neurodegenerative disease pathology by enhancing amyloid-β accumulation, promoting tau hyperphosphorylation, and disrupting hippocampal synaptic plasticity [34,35]. Some clinical investigations have reported that volatile anesthetic exposure may impair memory, attention, executive functions, and learning abilities, especially among elderly patients, but causality and risk magnitude have not been definitively established [36]. Furthermore, meta-analyses that compared different anesthetic techniques under certain clinical conditions have shown that inhalational anesthesia may be associated with a higher incidence of POCD compared to intravenous approaches [37]. While the precise mechanisms underlying these complications are not fully understood, current evidence strongly suggests that inhalational anesthetics exert complex biological effects that extend beyond the induction of reversible unconsciousness.

## 4. Neurotoxicity Induced by Inhalational Anesthesia

**Figure 1:**
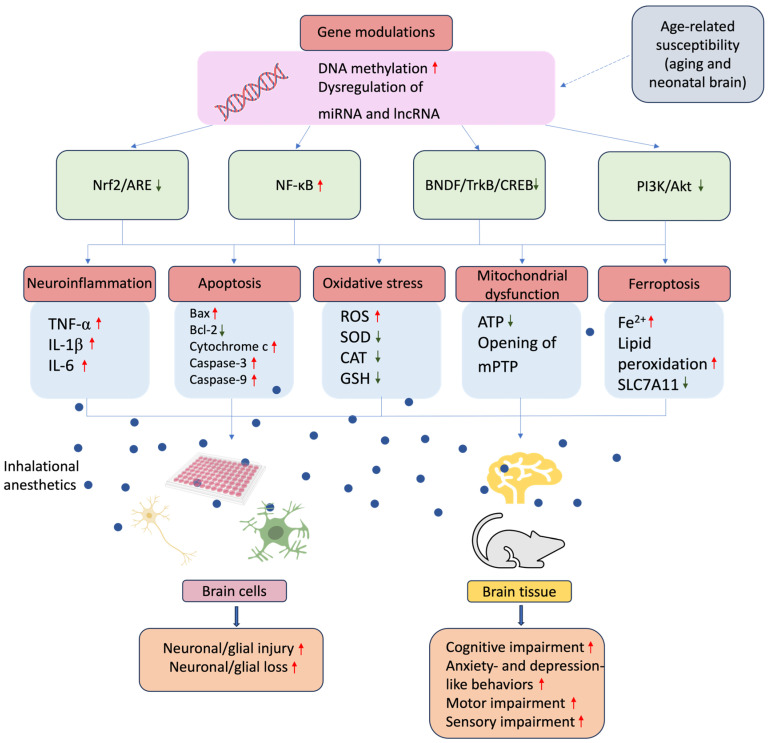
Neurotoxic mechanisms of inhalational anesthesia in brain tissue. ↑ denotes increase, ↓ denotes decrease. Green arrows denote beneficial effects, red arrows denote detrimental effects.

The strongest mechanistic evidence for inhalational anesthetics-induced neurotoxicity comes from studies in cell culture and animal models. A recurring theme is the anesthetic-induced activation of apoptotic pathways (Figure 1), excessive generation of reactive oxygen species (ROS), increased markers of inflammation, mitochondrial dysfunction, including collapse of the mitochondrial membrane potential, and impaired ATP production [38].

### 4.1. Apoptosis

Apoptosis, or programmed cell death, represents one of the most prominent and well-characterized mechanisms of inhalational anesthetic-induced neurotoxicity. Recent evidence indicates that during neurodevelopment, exposure to volatile anesthetics can trigger widespread neuronal apoptosis in the brain, particularly in areas undergoing active synaptogenesis, such as the hippocampus, cortex, and thalamus [39]. Studies suggest that inhalational anesthetics initiate the apoptotic cascade through the intrinsic (mitochondrial) pathway, characterized by mitochondrial outer membrane permeabilization, release of cytochrome c into the cytosol, formation of the apoptosome complex, and activation of executioner caspases such as caspase-3 [40]. Furthermore, experiments have demonstrated that exposure to volatile anesthetics decreases the expression of anti-apoptotic proteins, such as Bcl-2, and increases the expression of pro-apoptotic proteins, such as Bax, thereby shifting the balance toward apoptosis [41,42]. Aberrant activation of cyclin-dependent kinase 5 (CDK5) has emerged as a critical mediator of isoflurane-induced apoptosis. CDK5, which is normally involved in neuronal migration and synaptic plasticity, can become hyperactivated under pathological conditions, leading to phosphorylation of pro-apoptotic substrates and neuronal death [43]. In addition, the degradation of STAT3, a transcription factor involved in cell survival signaling, has been shown to contribute to isoflurane-induced neurotoxicity through calcineurin-mediated mechanisms [44].

### 4.2. Oxidative Stress

Oxidative stress, which results from an imbalance between ROS production and cellular antioxidant defense systems, also plays a role in inhalational anesthetics-induced neurotoxicity [45]. The mechanism of action of inhalation anesthetics on oxidative stress is that they increase ROS generation through mitochondrial dysfunction, disruption of the electron transport chain activity, and activation of NADPH oxidases. The brain is particularly vulnerable to oxidative damage during neurodevelopment due to its high metabolic rate, high lipid content, and relatively immature antioxidant defense systems [46]. Studies have shown that cellular response to oxidative stress involves the activation of antioxidant defense systems, including the Nrf2/ARE pathway, which regulates the expression of numerous antioxidant and cytoprotective genes [47]. However, in the context of inhalational anesthetics-induced neurotoxicity, these protective responses to oxidative stress are usually insufficient and cannot completely prevent neuronal damage. The role of specific genes involved in oxidative stress responses, including DJ-1/PARK7, SLC7A11, and OXR1, will be discussed in detail in the subsequent sections.

### 4.3. Mitochondrial Dysfunction

Recent studies have shown that mitochondria, which regulate cellular energy metabolism, calcium homeostasis, and apoptotic signaling, can be targets of inhalational anesthetics. Experiments demonstrated that inhalational anesthetics can induce loss of membrane potential, which is associated with the opening of the mitochondrial permeability transition pore (mPTP), allowing the release of cytochrome c and other pro-apoptotic factors from the intermembrane space into the cytosol, thereby leading to neuronal apoptosis [38]. Furthermore, inhalational anesthetics also impair mitochondrial respiratory chain function, leading to decreased ATP production and increased ROS production as byproducts of inefficient electron transport [48].

### 4.4. Neuroinflammation

Neuroinflammation, characterized by the activation of microglia and astrocytes, the release of pro-inflammatory cytokines, and the infiltration of peripheral immune cells, has emerged as an important mechanism contributing to anesthetics-induced neurotoxicity [49,50]. Multiple studies have documented microglial activation following anesthetic exposure, characterized by altered morphology, increased expression of activation markers, and enhanced phagocytic activity [51]. Recent investigations have revealed that complement C1q-mediated microglial synaptic elimination plays a critical role in sevoflurane-induced synaptic loss and cognitive impairment [52]. This process involves enhanced desialylation of synaptic proteins, making them targets for complement-mediated phagocytosis by microglia. In addition, astrocytes, the most abundant glial cells in the brain, respond to anesthetic exposure by altering gene expression and function. Upregulation of glutamate transporter 1 (GLT1) in astrocytes has been implicated in sevoflurane-induced inhibition of neurogenesis, potentially through excessive clearance of extracellular glutamate and disruption of glutamatergic signaling that is required for neural progenitor cell proliferation and differentiation [53]. The release of pro-inflammatory mediators, including high mobility group box 1 (HMGB1), TNF-α, IL-1β, and IL-6, contributes to neuronal damage through multiple mechanisms, including direct neurotoxicity, amplification of oxidative stress, and disruption of neurotrophic signaling [49]. The nuclear factor kappa B (NF-κB) signaling pathway is a key regulator of inflammatory gene expression and plays a central role in neuroinflammation induced by inhalational anesthetics [54].

### 4.5. Genetic Alterations

Inhalational anesthetics alter the expression of numerous protein-coding genes that play critical roles in neuronal survival, function, and development. In this narrative review, we examined evidence for alterations in specific genes associated with anesthetic neurotoxicity. We also explored the role of non-coding RNAs, including microRNAs (miRNAs) and long non-coding RNAs (lncRNAs), which have emerged as critical regulators of gene expression and play important roles in anesthetic-induced neurotoxicity [55]. These molecules do not encode proteins, rather they regulate gene expression at the post-transcriptional level through various mechanisms. In this review, we analyze the currently available studies on gene alterations induced by inhalation anesthesia, with a particular focus on neurotoxicity [56]. The goal of this work is to identify patterns of gene expression and correlate them with mechanisms underlying apoptotic, oxidative, inflammatory, and epigenetic changes. We did not identify unique gene signatures in the models studied, and the presence of inconsistent analytical procedures (mRNA vs. protein, early vs. late effects) further complicates data interpretation. Although anesthesia is necessary in clinical practice, our understanding of its molecular consequences is still limited. This review aims to summarize the current knowledge regarding genetic changes and neurotoxicity associated with inhalation anesthesia, identify promising directions for future research, and outline potential clinical applications.

In the pediatric population, concerns about anesthetic neurotoxicity have been intensified due to animal data showing widespread apoptotic neurodegeneration after exposure to anesthetics during critical periods of brain development [57]. In elderly patients, the relevance of anesthetic neurotoxicity is significant, since the advanced age, frailty, pre-existing cognitive impairment, vascular disease, sleep disturbance, and intraoperative physiologic instability all increase the likelihood of postoperative neurocognitive decline [58].

Understanding the mechanisms behind the neurotoxicity of inhalational anesthetics has practical significance that extends beyond laboratory neuroscience. Investigations into the use of inhalational anesthetics should help with drug selection, age-specific counseling, avoiding unnecessary exposure, prioritizing multimodal and regional strategies when appropriate, optimizing hemodynamic and oxygenation, and developing neuroprotective adjuncts.

## 5. Materials and Methods

A literature search was conducted in PubMed covering studies published in the last two decades. The search approach employed Boolean operators to link the following keywords: (“isoflurane” OR “sevoflurane” OR “desflurane” OR “nitrous oxide”) AND (“Neurotoxicity Syndromes” [Mesh] OR “neurotoxicity” OR “neurodegeneration” OR “synaptic plasticity” OR “apoptosis” OR “neuroinflammation” OR “neuroinflammation”) AND (“Gene Expression” [Mesh] OR “Genetics” [Mesh] OR “DNA Damage” [Mesh] OR “Epigenomics” [Mesh] OR “transcriptome” OR “mRNA” OR “microarray analysis” [Mesh] OR “RNA-seq” OR “qPCR” OR “Comet Assay” [Mesh] OR “reactive oxygen species” OR “oxidative stress” OR “antioxidant response element” OR “genotoxicity”) AND (“Neurons” [Mesh] OR “Neuroglia” [Mesh] OR “Astrocytes” [Mesh] OR “Microglia” [Mesh] OR “PC12 Cells” OR “SH-SY5Y” OR “U251” OR “H4 cells” OR “C6 glioma” OR “BV2 cells” OR “N2a cells” OR “Neuroblastoma” OR “Glioma” OR “primary culture”). Only original studies published in English and investigating genetic changes in anesthetic-associated neurotoxicity in animal or cell culture models were considered. Review articles, conference abstracts, editorials, and studies unrelated to neurotoxicity were excluded. Additional articles were obtained by screening the reference lists of the selected studies and relevant reviews.

## 6. Gene Alterations Associated with Sevoflurane-Induced Neurotoxicity

Studies from in vitro experiments involving neurons and glial cells show that sevoflurane consistently induces widespread changes in gene expression (Table 1) that affect multiple interconnected pathways associated with neurotoxicity, including oxidative stress, mitochondrial dysfunction, cell death mechanisms such as apoptosis and ferroptosis, neuroinflammation, and epigenetic alterations.

Across human neuronal and glial cell models, sevoflurane treatment consistently induces neurotoxicity through oxidative, apoptotic, inflammatory, and epigenetic mechanisms. In human hippocampal (HN-h) [59] and cortical neuronal (HCN-2) [60] cells, sevoflurane exposure reduces viability (app. 40–80% of control, depending on dose) while promoting apoptosis (app. 100-fold), oxidative stress, and inflammation, with these effects mediated by non-coding RNA regulatory systems, including upregulation of hsa-miR-302e that suppresses OXR1, disrupting redox homeostasis and amplifying oxidative stress, while increased lncRNA NKILA expression exacerbates neuronal damage through the miR-205-5p/ELAVL1 axis, further linking RNA dysregulation to apoptosis and inflammation [59,60]. In studies performed on human neuroblastoma cells (M17), sevoflurane-induced neurotoxicity was associated with mitochondrial dysfunction and oxidative stress, while simultaneously activating endogenous compensatory defense systems. Experimental investigations have found that elevated Sestrins-2 [61] and DJ-1 [39] expressions play protective roles against ROS accumulation, mitochondrial dysfunction, ATP depletion, and increased apoptosis (app. 4-fold). It is proposed that Sestrin-2 acts through p53-linked mitochondrial pathways, whereas DJ-1 preserves mitochondrial integrity, antioxidant capacity, and anti-apoptotic signaling. However, despite these compensatory adaptations, persistent sevoflurane exposure overwhelms cellular defenses, ultimately promoting Bax/caspase-mediated apoptosis and oxidative neuronal injury by increasing ROS (app. 2-fold) [39,61]. Furthermore, Tian et al. [62] and Liu et al. [54] showed that sevoflurane treatment of neuroglioma cells (H4) increased ROS generation, IL-6 expression (app. 2-fold), NF-κB activation, and the expression of apoptotic markers (Bax, caspase 3), while also upregulating the adaptive stress proteins Nrf2 (app. 2-fold) and HSP70 (app. 1.8-fold). Both Nrf2 and HSP70 have been shown to exert a partial cytoprotective effect, as pharmacologic enhancement of these pathways attenuates oxidative stress and apoptosis, but it is insufficient to fully counterbalance sevoflurane-induced pro-inflammatory and pro-apoptotic signaling, suggesting that oxidative stress-triggered inflammatory cascades are major contributors to human glial vulnerability.

In U251 astrocytic models, sevoflurane’s neurotoxic effects extend beyond oxidative-apoptotic paradigms by demonstrating engagement of ferroptosis-related pathways. Downregulation of SLC7A11 (app. 50–80%) and broader disruption of iron homeostasis genes (FPN1, FTH1, TFR1, NRF2, and HO-1) indicate that the susceptibility to ferroptosis is a major component of reduced cell viability (app. 40 to 80% of control, depending on dose and duration) [63]. Concurrently, sevoflurane induces alterations in the miR-211-5p/SIRT1/PI3K/Akt signaling pathway, further promoting mitochondrial dysfunction, autophagy dysregulation, and apoptosis (app. 4-fold), demonstrating that the astroglial responses to sevoflurane involve complex crosstalk among ferroptosis, mitochondrial apoptosis, and inflammation [63,64]. Neuroblastoma models using SH-SY5Y cells provide additional evidence that sevoflurane-induced human neuronal injury includes significant epigenetic remodeling and neurodegenerative molecular signatures. Specifically, sevoflurane suppresses METTL14-mediated m6A RNA methylation and downstream DUSP6 expression (app. 50% decrease), thus linking RNA modification directly to apoptotic vulnerability [65]. Simultaneously, Xu and colleagues showed that prolonged exposure (6 h) to sevoflurane dysregulates autophagic flux and increases BACE1 (app. 2-fold) and Aβ expressions (app. 1.7-fold), suggesting that sevoflurane may induce molecular changes resembling early neurodegenerative processes [66], indicating that its effects may extend beyond acute cellular damage and potentially contribute to long-term neuropathology.

Primary rodent neuronal and glial culture studies substantially expand the mechanistic framework that has been established in human cell models by demonstrating that sevoflurane-induced neurotoxicity is highly conserved across species while revealing additional developmental, ferroptotic, and neuroimmune aspects. In primary rat hippocampal and cortical neuronal cells, sevoflurane consistently reduces neuronal viability (app. 60–80% of control, depending of duration) and proliferation and activates classical apoptotic pathways, which are characterized by the increased expression of Bax (app. 1.7 to 9-fold), caspase-3 (app. 2 to 8-fold), and reduced Bcl-2 (app. 0.2 to 0.3-fold) expression [67,68,69,70,71,72]. Increased apoptosis (app. 4 to 7-fold, depending on dose and duration) is closely associated with oxidative stress (increased ROS and malondialdehyde (MDA) and depletion of superoxide dismutase (SOD) and glutathione (GSH)) and dysregulation of major antioxidant pathways such as Nrf2/ARE/HO-1. Multiple studies demonstrate that although sevoflurane may partially activate upstream Nrf2 signaling, its downstream antioxidant effectors, including HO-1, NQO1, GCL, and Prx1, are frequently suppressed, indicating dysfunctional antioxidant compensation rather than the effective cytoprotection [69,70,71]. Moreover, protective interventions aimed at targeting EPOR or restoring Nrf2 balance have been shown to attenuate oxidative stress, further supporting redox dysregulation as a central upstream driver of neuronal apoptosis [71].

Experiments conducted on rodent hippocampal cellular models also provide strong evidence that non-coding RNA systems are potential regulators of sevoflurane-induced neurotoxicity, as shown in recent studies where downregulation of miR-128-3p and lncRNA Rian, alongside increased NEAT1, expression, increases oxidative stress (app. 1.8-fold), inflammation, and apoptosis (app. 5 to 7-fold, depending on dose) through pathways involving NOVA1, LIMK1, and Nrf2 suppression [68,69,75]. These findings mirror observations in human neuronal lines and reinforce the concept that ncRNA-mediated gene dysregulation plays an important role in anesthetic neurotoxicity and that the restoration of these regulatory pathways significantly attenuates neuronal injury, underscoring their therapeutic relevance.

In primary fetal rat hippocampal neuronal cells, exposure to sevoflurane led to enhanced autophagy (app. 1.5-fold), which was mediated by dysregulated SIRT1/mTOR signaling and which appeared to accompany apoptotic progression (app. 2-fold) in immature hippocampal neurons [72]. Simultaneously, primary neonatal rat hippocampal neuronal cells exhibited significant DNA strand breaks, γ-H2AX accumulation, p53 activation, and direct suppression of SLC7A11, thereby linking sevoflurane exposure to genomic instability and ferroptosis via iron dysregulation and lipid peroxidation pathways [73]. These findings significantly extend the oxidative-apoptotic model by implicating DNA damage and ferroptosis as major contributors to developmental anesthetic neurotoxicity.

Another study conducted on primary mouse hippocampal neurons [74] further suggested that sevoflurane may induce molecular changes associated with long-term neurodegenerative susceptibility. Increased expression and fragmentation of ApoE (app. 6-fold), along with tau hyperphosphorylation, ATP depletion, reduced cell viability (app. 60% of control), oxidative stress, and inflammatory cytokine elevation after sevoflurane exposure may trigger Alzheimer-like neuropathological pathways under certain conditions, suggesting that repeated or prolonged anesthetic exposure could potentially extend beyond acute neurotoxicity and contribute to chronic neurodegeneration, particularly in vulnerable developmental phases.

Beyond direct neuronal injury, rodent glial cell studies highlight the importance of a critical secondary neuroimmune component. Specifically, sevoflurane robustly activates microglia and astrocytes, inducing increased IL-6 mRNA expression (app. 1.7 to 2.5-fold, depending on dose) [76], ERK signaling, PCSK9 upregulation (app. 3-fold), cGAS-STING activation, mitochondrial DNA release [77], and complement pathway activation (C1q/C3) [51]. These changes promote the release of inflammatory cytokines, synaptic phagocytosis, and maladaptive microglial activation, suggesting that sevoflurane-induced neurotoxicity may be substantially amplified through glial-mediated neuroinflammatory mechanisms. In particular, complement-associated synaptic pruning and the phagocytic transformation of microglia raise concerns regarding the long-term synaptic remodeling and cognitive dysfunction. PC12 models further demonstrate that TLR4/MyD88/NF-κB inflammatory signaling is a major mediator of sevoflurane-induced apoptosis (app. 4-fold) and inflammation, thereby reinforcing the centrality of neuroimmune dysregulation [78].

Although most studies demonstrated neurotoxic effects of sevoflurane, Chen et al. have shown that in an oxygen-glucose deprivation (OGD) model of hippocampal neuronal injury, sevoflurane postconditioning attenuated neuronal pyroptosis and inflammatory damage through an increase in the Mafb/DUSP14 signaling axis, resulting in suppression of NLRP3 inflammasome activation, suggesting that the biological effects of sevoflurane are highly context-dependent [79].

Animal studies conducted on maternal and early neonatal sevoflurane exposure showed that anesthetic administration during critical periods of neurodevelopment could disrupt fetal and neonatal brain maturation through interconnected mechanisms, including aberrant neuronal migration, neuroinflammation, ferroptotic susceptibility, and long-term behavioral dysregulation (Table 2). Unlike neonatal exposure animal models that primarily emphasize direct involvement of neuronal apoptosis, maternal exposure indicates that sevoflurane may first interfere with foundational developmental processes, thereby predisposing offspring to persistent neuropsychiatric and cognitive abnormalities.

Gestational exposure of rodents to maternal sevoflurane administration during embryonic cortical development disrupts interneuron migration and cortical circuits. Suppression of the CXCL12/CXCR4 signaling axis alters the orientation and distribution of migrating cortical interneurons, resulting in an abnormal excitatory/inhibitory balance, increased inhibitory synapse formation, and heightened susceptibility to epilepsy in adolescent offspring [80]. Furthermore, the authors showed an increase in anxiety- and depression-like behaviors, further suggesting that prenatal anesthetic exposure may transiently impair higher-order neurobehavioral programming during sensitive developmental periods. A study conducted on the same animal model identifies neuroinflammation as a major upstream mechanism underlying fetal anesthetic neurotoxicity. The results showed that the increased fetal brain IL-6 expression (app. 2-fold) following maternal sevoflurane exposure is sufficient to alter neuronal precursor cell proliferation in the subventricular zone, thereby disrupting normal neurogenesis and contributing to long-term learning deficits [76]. The absence of these abnormalities in IL-6 knockout mouse models implicates inflammatory cytokine signaling as a causal mediator rather than a secondary byproduct. Repeated neonatal exposure to sevoflurane further expands this inflammatory framework by demonstrating that postnatal sevoflurane exposure promotes sustained hippocampal neuroinflammation through SIRT1 suppression, NF-κB activation (app. 1.5-fold), and maladaptive microglial polarization (shift toward a pro-inflammatory M1 phenotype, characterized by increased CD86 and SOCS3 and reduced CD206) [81]. This neuroimmune imbalance is strongly associated with hippocampus-dependent cognitive impairment, suggesting that microglial dysfunction may be a major contributor to memory deficits. Additional gestational studies broaden the mechanistic landscape by implicating astrocytic dysfunction and ferroptosis-related pathways. Maternal sevoflurane exposure during embryonic stages suppresses astrocyte proliferation and activation, reduces GFAP and Ki67 expressions, and enhances inflammatory cytokine production in offspring hippocampi [63]. Downregulation of SLC7A11 (app. 0.1-fold) indicates that impaired antioxidant and iron-regulatory systems may predispose the developing brain to ferroptotic injury. This introduces ferroptosis as a potentially underrecognized mechanism of developmental anesthetic neurotoxicity, complementing established inflammatory and migratory abnormalities.

Neonatal rodent studies identify apoptosis as one of the earliest and most consistent pathological consequences of sevoflurane exposure during the critical neurodevelopmental period. Multiple mouse and rat models demonstrate that sevoflurane induces widespread hippocampal and cortical apoptosis, as evidenced by increased cleaved caspase-3, elevated Bax/Bcl-2 ratios, DNA fragmentation, and neuronal structural degeneration [41,82,83,84]. These apoptotic effects are accompanied by suppression of essential neurodevelopmental survival pathways, including BDNF/TrkB, cAMP/CREB, PI3K/Akt/mTOR [82], DISC1 [84], and IGF1R [41] signaling, indicating that sevoflurane interferes with fundamental mechanisms of neuronal maturation, synaptic plasticity, and survival. Studies involving ENSMUST00000136025/Bim [83], miR-96/IGF1R [41], and Rian-associated neuroprotection [75] further demonstrate that apoptotic vulnerability is closely linked to broader transcriptional and post-transcriptional regulatory disturbances. These findings suggest that neonatal sevoflurane exposure impairs neuronal viability not only through direct cytotoxicity but also by destabilizing critical developmental signaling in these animals.

Neuroinflammation represents another key mechanism underlying neonatal sevoflurane neurotoxicity, as demonstrated by elevated levels of hippocampal IL-1β, IL-18, IL-6, and TNF-α, alongside NLRP3 inflammasome activation (app. 4-fold mRNA increase), pyroptotic signaling, oxidative stress, and microglial dysregulation [85,86,87]. Specifically, the H19/miR-152-3p/USP30 signaling pathway has been implicated in promoting microglial activation and inflammatory injury [85], while the suppression of MEG3 contributes to NLRP3-mediated pyroptosis [86]. Additionally, miR-27a-3p/PPARγ dysregulation of oxidative stress and inflammation results in hippocampal damage [87]. Collectively, these studies indicate that neuroinflammation functions not only as a secondary response to anesthetic-induced injury but also as an active driver of developmental neuropathology.

Epigenetic and non-coding RNA dysregulation has emerged as an important mediator of neonatal anesthetic neurotoxicity, thereby expanding the mechanistic framework beyond traditional apoptosis-centered models. Sevoflurane exposure consistently alters multiple lncRNAs, miRNAs, and methylation-dependent pathways, including H19/USP30 [85], MEG3/NLRP3 [86], Rian [75], Rik-203/miR-101a-3p/GSK-3β [88], miR-96/IGF1R [41], miR-27a-3p/PPARγ [87], and widespread lncRNAs/mRNAs transcriptional remodeling [90]. Observations of reductions in global neuronal DNA methylation, hypomethylation of the Arc promoter, and evidence of transgenerational epigenetic inheritance strongly suggest that sevoflurane can induce heritable neurodevelopmental alterations [91]. Neonatal anesthetic exposure may permanently reshape the developmental gene regulatory architecture, potentially influencing both immediate neuronal outcomes and future cognitive susceptibility, including effects on subsequent generations.

In addition to apoptosis, inflammation, and epigenetic remodeling, Jiang et al. showed that repeated exposure disrupts long-chain fatty acid metabolism, suppresses PPARβ (app. 0.5-fold) signaling, impairs oligodendrocyte maturation, reduces myelin basic protein expression, and induces hypomyelination [90]. TYMS/ERMN dysregulation (reduced app. 0.5-fold and 0.2-fold, respectively) links folate metabolism impairment to defective myelination and cognitive decline, findings conserved in both neonatal mice and rhesus macaques [89]. These studies demonstrate that sevoflurane significantly affects broader neurodevelopmental infrastructure, including white matter formation, metabolic programming, and myelin integrity. Additional studies in primates reinforce translational relevance by demonstrating conserved disruptions in lipid metabolism, inflammatory cytokine profiles, oxidative stress markers, and neuronal viability [92]. Collectively, these mechanistic findings indicate that neonatal sevoflurane neurotoxicity extends beyond isolated neuronal death, encompassing systemic disturbances in neurodevelopmental architecture, metabolic homeostasis, and structural brain maturation.

Research conducted on adult mice and rat models demonstrates that sevoflurane induces apoptosis (app. 4-fold) and neuroinflammation in animals with mature nervous systems (Table 3). Consistent with findings in the PC12 cellular model [78], sevoflurane-induced downregulation of miR-424 and subsequent activation of the TLR4/MyD88/NF-κB pathway have been confirmed in the hippocampus of adult rats. These molecular changes are associated with the elevated levels of inflammatory markers (TNF-α, IL-1β, and IL-6), reduced levels of the anti-inflammatory cytokine IL-10, and increased pro-apoptotic markers (Bax and caspase-3). In the same model, Su et al. [93] reported increased inflammatory cytokines and apoptotic factors, along with downregulation of MiR-410-3p and upregulation of C-X-C motif chemokine receptor 5 (CXCR5). The lentiviral-mediated overexpression of MiR-410-3p decreased CXCR5 expression, indicating that MiR-410-3p inhibits CXCR5 and mitigates sevoflurane-induced neurotoxicity. Similarly, adult mice exposed to sevoflurane exhibited concentration-dependent increases in inflammatory markers (IL-6 (app. 1.8 to 2.2-fold), IL-10 (app. 1.8 to 2-fold), and TNF-α (app. 1.9 to 2.2-fold) and apoptosis [94]. In these mice, the lncRNA Gm5106 and the transcription factor Hoxa5 were significantly upregulated, while miR-27-3p expression was downregulated. The silencing of Gm5106 suppressed inflammatory markers and increased miR-27-3p expression, implicating Gm5106 in sevoflurane-induced neuroinflammation [94]. Deng et al. [65] found that adult mice treated with sevoflurane exhibited reduced expression of m6A, METTL14, and DUSP6, as previously observed in SH-SY5 cells, and these changes were associated with cognitive deficits. The overexpression of METTL14 alleviated these impairments, increased m6A levels, and upregulated DUSP6, suggesting that METTL14 regulates DUSP6 expression via m6A methylation. In contrast to these studies, sevoflurane preconditioning of adult rats has been shown to attenuate cerebral ischemia/reperfusion-induced neuroinflammation by suppressing ROS-dependent activation of the NLRP3 inflammasome [95]. Also, Peng et al. demonstrated that in a cerebral ischemia–reperfusion model conducted on mice, sevoflurane post-conditioning activated the Shh-YAP1 signaling pathway, preserved mitochondrial function, and attenuated apoptosis in the brain [96]. Although these findings originate from an ischemia/reperfusion model and not from direct anesthetic neurotoxicity, they demonstrate that the effects of sevoflurane are context-dependent.

Experimental models of aged rats reveal that sevoflurane inhalation anesthesia induces extensive neurotoxic effects through diverse molecular, genetic, epigenetic, and cellular disruptions, ultimately compromising neuronal integrity and cognitive function. A consistent pathological feature is the induction of oxidative stress, evidenced by excessive ROS production, increased NADPH oxidase 2 (NOX2) expression, elevated MDA levels, and depletion of endogenous antioxidants such as SOD, GSH, catalase (CAT), and MnSOD [54,62,97,98,99]. This persistent redox imbalance leads to mitochondrial dysfunction, lipid peroxidation, neuronal apoptosis, autophagy, and structural disorganization in hippocampal tissue, resulting in significant impairments in spatial learning and memory. At the molecular level, sevoflurane exposure markedly suppresses neuroprotective pathways, particularly by downregulating brain-derived neurotrophic factor (BDNF) and attenuating PI3K/Akt/mTOR signaling, which reduces neuronal survival, synaptic plasticity, and adaptive resilience mechanisms essential for cognitive preservation [97]. Concurrently, oxidative injury activates the Nrf2 antioxidant defense system in a temporally dynamic and context-dependent manner. Although acute oxidative stress induced by sevoflurane may initially upregulate Nrf2 as a protective response [62], prolonged exposure, age-related vulnerability, and pathological factors often impair downstream Nrf2/ARE/HO-1 signaling, resulting in an inadequate antioxidant defense despite upstream activation [69].

In addition to the oxidative and apoptotic injury, recent transcriptomic and epigenetic studies demonstrate that sevoflurane significantly alters global gene expression networks in the aging hippocampus. Comprehensive whole-transcriptome microarray analyses conducted on aged rats identified over 25,000 lncRNAs, with 514 significantly dysregulated after sevoflurane exposure, including 232 upregulated and 282 downregulated transcripts. Notably, the Sancr family (Sancr1–4) exhibited consistent alterations, indicating extensive epigenetic remodeling in response to inhalational anesthesia [98]. Functional analyses linked these differentially expressed lncRNAs and their target genes, such as Hif3a, Prkcd, and Nfe2l2, to pathways involved in mitochondrial dysfunction, oxidative stress, metabolic dysregulation associated with aging, DNA damage, apoptosis, adipocytokine signaling, MAPK pathway activation, and broader neurodegenerative processes. Among these lncRNAs, NEAT1 [69] has emerged as a key pathological regulator since its overexpression suppresses Nrf2/ARE/HO-1 signaling, thereby worsening oxidative stress, mitochondrial dysfunction, neuronal apoptosis, and cognitive decline [62].

Proteostasis and inflammatory pathways play significant roles in modulating injury severity. The sevoflurane-induced upregulation of HSP70 (app. 1.5-fold) likely reflects an endogenous compensatory response to stress. The pharmacological inhibition of HSP90 with 17AAG further increases HSP70 expression and markedly reduces oxidative stress, apoptosis, IL-6 production, and NF-κB-mediated inflammatory signaling [54]. Similarly, interventions such as minocycline, which enhance Nrf2-mediated antioxidant defenses, demonstrate the therapeutic potential of targeting redox-sensitive and inflammatory pathways [62].

Guo et al. [99] demonstrated that both the frequency and cumulative duration of sevoflurane exposure significantly affect the long-term neurological outcomes in aged rats. Their study showed that while both single and repeated exposures impaired learning and memory, repeated exposure resulted in greater and more persistent deficits, even at concentrations that were less harmful when administered once. Specifically, repeated exposure to 2.5% sevoflurane led to a significant reduction in hippocampal BDNF protein levels, increased NF-κB mRNA expression, and elevated hippocampal neuronal apoptosis (app. 28-fold), thereby linking cumulative anesthetic exposure to progressive neurotrophic suppression, inflammatory activation, and structural neuronal injury. These results indicate that repeated anesthetic administration may accelerate neurodegenerative processes beyond those observed with isolated exposures in aged animals. Whether these findings might pose a clinically relevant concern for elderly patients undergoing multiple procedures remains to be determined.

Further investigation into inflammatory mechanisms conducted by Li et al. [100] identified the voltage-gated potassium channel Kv1.3 (app. 1.5-fold) as a novel contributor to sevoflurane-induced cognitive dysfunction. In aged mice, sevoflurane exposure significantly increased hippocampal Kv1.3 expression, primarily in activated microglia, which promoted neuroinflammatory responses and cognitive decline. Pharmacological inhibition of Kv1.3 alleviated learning and memory impairments, reduced neuronal injury, suppressed NLRP3 inflammasome activation, and shifted the transition of microglia from pro-inflammatory M1 to anti-inflammatory M2 phenotype.

Additional evidence presented by Zhang et al. [101] utilized single-nucleus RNA sequencing in aged marmosets and mice to characterize cell-specific transcriptomic responses to prolonged sevoflurane exposure. Following the six hours of sevoflurane anesthesia, FKBP5 expression was significantly increased in the hippocampus of both aged marmosets and mice, with the highest expression observed in microglia. This upregulation was confirmed by Western blot analysis, and the microglia-specific FKBP5 conditional knockout improved neurocognitive performance after sevoflurane exposure or surgery. Transcriptomic sequencing further revealed that FKBP5 was primarily associated with inflammatory signaling pathways, including chemokine and cytokine–cytokine receptor-mediated signaling.

## 7. Gene Alterations Associated with Isoflurane-Induced Neurotoxicity

Studies examining the effects of isoflurane in vitro have shown that its neurotoxic effects are similar to those previously described for sevoflurane. In human-derived glial and neuronal cell models, isoflurane consistently disrupts key pathways involved in neuroinflammation, cell survival, and oxidative stress, thereby creating a complex neurotoxic environment (Table 4). Zhang et al. [102] showed that in H4 cells, isoflurane exposure increased Bax (app. 1.7-fold) and reduced Bcl-2 (app. 0.6-fold) expression, and increased ROS production (app. 1.7-fold), resulting in increased apoptosis (app. 6-fold). Furthermore, Yang et al. [103] found that isoflurane impairs vital pro-survival signaling by destabilizing STAT3, reducing its phosphorylation, and depleting anti-apoptotic proteins such as Bcl-xL, survivin, and MnSOD in U251 cells. This disruption weakens antioxidant defenses and increases vulnerability to apoptosis and oxidative stress. Overall, these findings indicate that isoflurane triggers apoptosis while concurrently weakening protective signaling pathways, leading to a combined effect that heightens cellular susceptibility.

Studies conducted on SH-SY5Y cells [40,45,104] showed that oxidative stress emerges as a central mediator of neuronal injury. Multiple studies demonstrated that isoflurane significantly reduced cell viability (app. 60–80% of control, depending on duration) by increasing ROS accumulation (app. 2.5-fold), suppressing antioxidant enzymes (SOD and CAT), and elevating lipid peroxidation markers (MDA). These biochemical disturbances were consistently accompanied by the activation of apoptosis (app. 8-fold), including increased caspase-3/7 activity and an altered Bcl-2/Bax ratio. Notably, the cells responded to isoflurane by activating endogenous defense mechanisms, including the upregulation of the Nrf2/ARE [45] antioxidant pathway and increased DJ-1 expression [40], both of which appear to function as compensatory survival responses aimed at mitigating oxidative injury. However, despite the activation of these protective systems, oxidative stress and mitochondrial dysfunction persisted, indicating that these adaptive responses are insufficient to fully neutralize isoflurane-induced damage. The exacerbation of toxicity following DJ-1 silencing further reinforces the protective importance of these endogenous mechanisms while simultaneously underscoring the severity of isoflurane’s oxidative burden. In addition to inflammatory and oxidative mechanisms, emerging evidence indicates that isoflurane may also exert neurotoxic effects through epigenetic and post-transcriptional dysregulation. Wu et al. [104] demonstrated that isoflurane downregulated miR-214 expression in SH-SY5Y cells, resulting in increased PTEN activity and subsequent inhibition of the PI3K/Akt survival pathway. This molecular alteration further increased oxidative stress, apoptosis, and neuronal injury, while the restoration of miR-214 expression effectively reversed these neurotoxic effects.

Studies using neuronal cell models demonstrated that isoflurane exposure induced neurotoxic effects by increasing oxidative stress, mitochondrial dysfunction, apoptosis, synaptic impairment, and the dysregulation of survival-associated signaling pathways. Bai et al. [105] reported that isoflurane exposure inhibited neuronal cell proliferation and induced significant DNA damage, which was accompanied by the increased numbers of apoptotic cells (app. 10x increase) and elevated activities of caspases-3, -8, and -9. These changes were associated with severe mitochondrial dysfunction, including a loss of mitochondrial membrane potential, increased opening of mPTP, elevated ROS production, reduced CAT and SOD activities, ATP depletion, and disturbances in intracellular calcium homeostasis. Similarly, Xie et al. [106] demonstrated in primary cortical neurons that isoflurane exposure significantly reduced neuronal viability (app. 30% of control) and synaptic integrity, as indicated by decreased expression of Synapsin I and PSD95, along with excessive ROS generation (app. 3.5-fold). The neurotoxic effects were also accompanied by reduced LanCL1 expression, whereas adenoviral overexpression of LanCL1 restored synaptic growth, reduced oxidative stress, and improved neuronal survival. These findings suggest a critical antioxidant and neuroprotective role for LanCL1 during anesthesia-induced neuronal injury.

Research utilizing hippocampal neuronal models has further emphasized the significance of apoptosis-related and miRNA-mediated signaling pathways in isoflurane neurotoxicity. Li et al. [107] demonstrated that isoflurane exposure in hippocampal neurons upregulated miR-302b-3p and induced neuronal injury, as evidenced by increased Bax and calpain expression, reduced Bcl-2 levels, decreased cell viability (0.38-fold), elevated lactate dehydrogenase (LDH) release, and enhanced apoptosis (app. 5-fold). The silencing of miR-302b-3p significantly mitigated these detrimental effects, whereas its overexpression exacerbated neuronal injury. Mechanistically, PTEN was identified as a direct downstream target of miR-302b-3p, thereby linking isoflurane-induced neurotoxicity to dysregulation of PTEN/AKT signaling pathways. Similarly, Wang et al. [43] showed that isoflurane induced aberrant CDK5 activation in cultured hippocampal neurons derived from embryonic Sprague Dawley (SD) rats through calpain-mediated cleavage of p35 into p25. This process resulted in altered CDK5 localization, suppression of myocyte enhancer factor 2 (MEF2) signaling, increased phospho-MEF2A-Ser-408 expression, and marked neuronal apoptosis (app. 5-fold) as confirmed by flow cytometry and TUNEL staining. Pharmacological inhibition of CDK5 with roscovitine or transfection with dominant-negative CDK5 substantially reduced apoptosis and restored neuronal survival signaling, thereby highlighting CDK5 dysregulation as a major contributor to developmental anesthesia-induced neurotoxicity. In HT22 neuronal cells, isoflurane similarly reduced viability (app. 50% of control), induced oxidative stress, mitochondrial dysfunction, apoptosis (app. 8-fold), and suppression of miR-214 expression, while simultaneously activating PTEN and inhibiting Akt-mediated survival signaling [108]. However, Klenke et al. [112] found that isoflurane exposure did not significantly alter the methylation patterns of apoptosis- or cytokine-related genes in HT22 cells, suggesting that this model may have limited utility for investigating the epigenetic mechanisms associated with inhalational anesthetic exposure.

Studies employing glial and microglial cell models consistently demonstrate that isoflurane exposure induces a robust inflammatory and oxidative response. Hirotsu et al. [76] found that isoflurane significantly increased IL-6 mRNA expression in BV-2 microglial cells and primary cultured astrocytes, with no effect in neuronal cells, indicating a central role for glial activation in anesthesia-associated neuroinflammation. Similarly, exposure of BV2 microglial cells to isoflurane resulted in reduced viability (app. 60% of control) increased ROS production (app. 3.3-fold), reduced SOD activity, enhanced expression of TNF-α and IL-1β, increased cyclooxygenase-2 (COX-2) and prostaglandin E2 (PGE2) production, elevated ionized calcium-binding adapter molecule 1 (Iba1) expression, and reduced triggering receptor expressed on myeloid cells 2 (TREM2) expression, collectively indicating oxidative injury and pronounced microglial activation [109]. Furthermore, Jiang et al. [110] demonstrated that isoflurane exposure significantly increased apoptosis (app. 4-fold) and expression of pro-inflammatory cytokines, including IL-1β, TNF-α, IL-6, and IL-8 in BV2 cells, while simultaneously promoting the expression of both M1-associated markers (CD16 and inducible nitric oxide synthase (iNOS)) and M2-associated markers (CD206 and arginase 1 (Arg1). The study also identified upregulation of toll-like receptor 4 (TLR4) signaling following isoflurane exposure, suggesting that the activation of TLR4-mediated inflammatory pathways may be a key mechanism underlying microglial activation and neuroinflammatory injury induced by inhalational anesthetics.

Experiments conducted on PC12 cells further confirmed the significance of oxidative stress and miRNA dysregulation in isoflurane-induced neurotoxicity. One study demonstrated that the downregulation of miRNA-153 significantly increased apoptosis, suppressed cell growth, enhanced oxidative stress, reduced nuclear factor erythroid 2–related factor 2 (Nrf2)/heme oxygenase-1 (HO-1) signaling, and increased Bax expression. In contrast, the overexpression of miRNA-153 attenuated oxidative injury, promoted neuronal survival, increased SOD and catalase (CAT) activities, and reduced MDA and myeloperoxidase (MPO) levels. The activation of the Nrf2/ARE pathway by dimethyl fumarate partially reversed the detrimental effects of anti-miRNA-153 treatment, supporting the involvement of miRNA-153/Nrf2 signaling in cytoprotection against isoflurane-induced neuronal injury [47]. Similarly, another study using PC12 cells demonstrated that isoflurane exposure significantly reduced cell viability (app. 50% of control) and increased apoptosis (app. 3-fold), while simultaneously elevating TNF-α, IL-6, and ROS levels (app. 3.5-fold) and decreasing GSH and SOD activity. Isoflurane also markedly reduced miR-409 expression, further supporting the concept that oxidative stress, inflammation, and miRNA dysregulation collectively contribute to inhalational anesthetic-induced neurotoxicity in neuronal cell models [111].

In contrast to these findings, Cao et al. [113] demonstrated that isoflurane preconditioning increased miR-203 expression in neuron-like cells (B35), thereby enhancing resistance to oxygen-glucose deprivation-induced injury. This protective effect was associated with increased Akt phosphorylation, suggesting the activation of pro-survival signaling pathways, indicating that the effects of isoflurane may vary according to experimental conditions and underlying cellular stressors.

Neurodevelopmental models are among the most important preclinical approaches for investigating inhalational anesthetic-induced neurotoxicity, primarily because the immature brain is highly susceptible to apoptosis, neuroinflammation, oxidative stress, synaptic dysfunction, and long-term cognitive impairment (Table 5). Studies in neonatal rodents and fetal models consistently show that early isoflurane exposure induces widespread molecular and genetic alterations, structural neuronal damage, and behavioral deficits. These investigations increasingly focus on identifying dysregulated inflammatory mediators, apoptotic pathways, miRNAs, and intracellular survival signaling mechanisms associated with the anesthesia-induced developmental neurotoxicity.

Among the earliest observed alterations, Hirotsu et al. [76] demonstrated that maternal isoflurane exposure significantly increased IL-6 mRNA expression in fetal mouse brains, suggesting the activation of neuroinflammatory pathways during prenatal development. Several subsequent studies identified apoptosis as a central mechanism underlying neonatal anesthetic-induced injury. Yong et al. [114] showed that repeated neonatal isoflurane exposure induced extensive neuronal apoptosis (app. 5-fold) and synaptic loss in the cerebral cortex, hippocampus, amygdala, and hypothalamus, accompanied by reduced expression of PPAR-α and Bcl-2 at both mRNA and protein levels. Importantly, treatment with compound 21 attenuated apoptotic injury and restored PPAR-α and Bcl-2 expression, suggesting the activation of anti-apoptotic signaling pathways. Similarly, Wang et al. [43] demonstrated that isoflurane induced aberrant activation of CDK5 through calpain-mediated cleavage of p35 into p25, leading to increased Bax and cleaved caspase-3 expression, suppression of Bcl-2 and MEF2 signaling, and subsequent learning and memory deficits. Pharmacological inhibition of CDK5 with roscovitine significantly alleviated both molecular and behavioral abnormalities. Furthermore, isoflurane-induced neurotoxicity was also associated with the suppression of the STAT3 survival pathway through calcineurin activation (app. 2-fold), resulting in reduced survival and Bcl-xl expression and enhanced neuronal apoptosis. FK506 attenuated these effects, further emphasizing the role of disrupted intracellular survival signaling in developmental anesthetic neurotoxicity [103].

Recent investigations have also highlighted the importance of miRNA dysregulation in mediating anesthesia-induced neuronal injury. Yang et al. [115] reported that neonatal isoflurane exposure reduced miR-124 expression while increasing EGR1 levels in hippocampal tissues, leading to impaired spatial learning, decreased neuronal viability (app. 25% of control), and enhanced apoptosis (app. 3-fold). The restoration of miR-124 expression improved cognitive performance and attenuated neuronal injury by suppressing EGR1 signaling. Likewise, Li et al. [107] identified the miR-302b-3p/PTEN axis as another important regulator of isoflurane-induced neurotoxicity, demonstrating that the silencing of miR-302b-3p alleviated neuronal injury and spatial memory deficits by upregulating PTEN.

Furthermore, neuroinflammatory and oxidative mechanisms were also consistently implicated in neonatal anesthesia-induced injury. Sun et al. [116] demonstrated that isoflurane exposure increased levels of NF-κB, TNF-α, IL-1β, and oxidative stress markers and induced neuronal apoptosis (app. 6.5-fold), neurological deficits, and cognitive impairment. Similarly, Jiang et al. [110] showed that isoflurane activated TLR4/MyD88/TRAF6 and downstream p38 MAPK/NF-κB signaling pathways, accompanied by increased pro-inflammatory cytokine expression, apoptosis (app. 4-fold), and microglial activation. Also, genistein treatment attenuated neuroinflammation, promoted M2 microglial polarization, and reduced neuronal apoptosis, indicating a potential protective effect against anesthesia-induced inflammatory injury. In addition to apoptotic and inflammatory mechanisms, prolonged neonatal isoflurane exposure impaired synaptic integrity, as evidenced by reduced Synapsin I and PSD95 expression, decreased LanCL1 levels, and increased ROS production in neonatal mouse cortices. Notably, LanCL1 overexpression restored synaptic protein expression, reduced oxidative stress, and improved behavioral outcomes, suggesting an important neuroprotective role against anesthesia-induced synaptic dysfunction [106].

Studies conducted on mature animal models further confirmed the evidence that inhalational anesthetics, particularly isoflurane, can produce significant neurotoxic effects beyond the vulnerable neurodevelopmental period (Table 6). These studies have shown that anesthesia-associated cognitive dysfunction in mature animals is closely linked to neuronal apoptosis, neuroinflammation, oxidative stress, and dysregulation of multiple miRNA-dependent signaling pathways. In addition, many of these studies highlighted the importance of endogenous cytoprotective mechanisms and identified several potential therapeutic targets to attenuate anesthesia-induced neuronal injury.

Shao et al. [47] demonstrated that isoflurane anesthesia significantly downregulated miRNA-153 expression in hippocampal tissues in vivo, accompanied by marked increases in caspase-3 and caspase-9 activities, thereby inducing neuronal apoptosis. The study suggested that the suppression of miRNA-153 contributes to isoflurane-induced neurotoxicity by disrupting cellular survival pathways, while the Nrf2/ARE signaling cascade appears to play an important compensatory cytoprotective role against oxidative and apoptotic injury. The authors emphasized that acute exposure to isoflurane can damage the hippocampus and impair learning and memory. Similarly, Qi et al. [111] reported findings that isoflurane exposure significantly impaired spatial learning and memory in rats, as evidenced by prolonged escape latency periods during Morris water maze reversal testing. These behavioral abnormalities were accompanied by increased hippocampal neuronal apoptosis (app. 4.5-fold) and reduced miR-409 expression in hippocampal tissues, further supporting the concept that miRNA dysregulation is an important mechanism underlying anesthesia-associated neurotoxicity in adult animals. Consistent with these observations, Si et al. [117] showed that isoflurane-induced cognitive dysfunction was also strongly associated with the activation of neuroinflammatory pathways. Rats exposed to isoflurane showed impaired learning and memory performance, and elevated levels of pro-inflammatory cytokines (IL-1β, IL-6, and TNF-α) in the hippocampus. Further investigation showed that hippocampal miR-212-5p expression was markedly reduced following isoflurane exposure, whereas agomir administration upregulated miR-212-5p, substantially improving behavioral outcomes and suppressing inflammatory cytokine production. This data highlighted the neuroprotective and anti-inflammatory properties of miR-212-5p and could represent a promising therapeutic strategy to reduce neuronal injury [117]. In contrast to studies reporting neurotoxicity, isoflurane has demonstrated neuroprotective effects on mice and rats in ischemia–reperfusion models by reducing infarct size, apoptosis, and neuroinflammation through inhibition of NF-κB and IL-1β signaling, suggesting that the effects of isoflurane may be context-dependent [118].

Studies performed on aged animal models further demonstrated that the mature brain may be particularly susceptible to inhalational anesthetic-induced neurotoxicity and POCD. Zhang et al. [119] identified the mechanisms underlying isoflurane-induced POCD in aged rats and the PYPAF1 inflammasome as an important contributor to anesthesia-associated neurotoxicity. Exposure to 2% isoflurane resulted in significant cognitive impairment accompanied by increased PYPAF1 and ASC expression, microglial activation, elevated IL-1β and IL-18 levels, enhanced caspase-1 activity, and increased neuronal apoptosis (app. 8.5-fold) in hippocampal tissues. Importantly, lentivirus-mediated silencing of PYPAF1 partially reversed these pathological changes, attenuating neuroinflammation, reducing neuronal apoptosis, and improving cognitive performance, thereby suggesting that inflammasome activation plays a central role in isoflurane-induced POCD in aged animals. Also, Li et al. [120] showed that glycogen synthase kinase-3β (GSK-3β) was involved in isoflurane-induced neuroinflammation and cognitive impairment in aged rats. Isoflurane exposure significantly increased GSK-3β expression in the hippocampus, along with enhanced nuclear acetyl-NF-κB p65 activation and elevated levels of pro-inflammatory cytokines (TNF-α, IL-1β, and IL-6). Notably, prophylactic lithium chloride treatment, a non-selective GSK-3β inhibitor, substantially attenuated these inflammatory alterations and improved cognitive outcomes, further stressing the importance of inflammatory signaling pathways in anesthesia-associated cognitive decline in aging brains. Consistent with these findings, Qi et al. [42] demonstrated that isoflurane exposure disrupted neuronal calcium homeostasis in aged rats by significantly increasing intracellular calcium concentrations in cortical slices and hippocampal CA1 neurons. These changes were associated with increased apoptosis (app. 5-fold), increased Bax and decreased Bcl-2 expression (an elevated Bax/Bcl-2 ratio), and impaired hippocampal synaptic function, thus producing memory and learning deficits. Overall, studies conducted in aged models consistently indicate that neuroinflammation, apoptotic signaling, calcium dysregulation, and inflammasome activation represent major mechanisms underlying inhalational anesthetic-induced cognitive dysfunction in the aging brain and suggest that age-associated vulnerability, together with pre-existing inflammatory and degenerative changes, may substantially increase the detrimental effects of inhalational anesthetics on the central nervous system.

**Table 6:** Gene alterations associated with isoflurane-induced neurotoxicity in adult rodents.

Reference	Specific Model	Anesthetic Protocol	Genes Alterations	Neurotoxic Event
***Adult models***				
[47]	Adult mice (C57BL/6J)	1.4% for 2 h	miR-153 ↓	Apoptosis ↑
[111]	Adult rats (SD)	1.4% for 2 h	miR-409 ↓	Apoptosis ↑ Cognitive impairment ↑
[117]	Adult rats (SD)	3% for 6 h	miR-212-5p ↓	Neuroinflammation ↑ Cognitive impairment ↑
***Aged models***				
[119]	Aged rats (SD)	2% for 4 h	PYPAF1 ↑ ASC ↑ IL-1β ↑ IL-18 ↑	Apoptosis ↑ Neuroinflammation ↑ Cognitive impairment ↑
[120]	Aged rats (SD)	1.4% for 6 h	GSK-3β ↑ acetyl-NF-κB p65 ↑ TNF-α ↑ IL-1β ↑ IL-6 ↑	Neuroinflammation ↑ Cognitive impairment ↑
[42]	Aged rats (SD)	1.8% for 2 h + surgery	Bcl-2 ↓ Bax ↑	Apoptosis ↑ Cognitive impairment ↑

An upward arrow denotes increased values for the estimated parameter; a downward arrow denotes decreased values for the estimated parameter.

Overall, the evidence obtained from the analysis of the preclinical data indicates that the neurotoxicity of inhalational anesthetics such as sevoflurane and isoflurane is associated with the multilayered interaction of different molecular mechanisms, including apoptosis, oxidative stress, mitochondrial dysfunction, neuroinflammation, ferroptosis, and epigenetic reprogramming, such as DNA methylation changes and altered folate metabolism. The pro-apoptotic effects, including changes in the gene expression of the Bax/Bcl-2 ratio and the activation of caspase 3/9 and release of cytochrome c, as well as increased expression of NF-κB, IL-6, and TNF-α leading to neuroinflammation, are con-istently demonstrated by both cell and animal models. Dysregulation of non-coding RNAs (e.g., miRNAs and lncRNAs) also seems to be a significant modulator in both anesthetic models, influencing apoptotic, inflammatory, and oxidative stress-response pathways. Animal models, which demonstrated age-related susceptibility, further provide a unique mechanistic insight into region-specific neuronal apoptosis, microglial activation, persistent neuroinflammation, cognitive impairment (decreased BDNF), and myelination deficits (sensory and motor impairments), emphasizing the potential translational relevance of these findings. Moreover, there are some contradictory findings; specifically, in certain studies, the adaptive responses are sometimes observed, such as an increased antioxidant gene expression (e.g., Nrf2/ARE pathway [45,62]), but they are often inadequate to prevent injury, particularly during vulnerable periods of brain development. In contrast, other studies have reported Nrf2 and its downstream targets to be suppressed or not well activated, resulting in insufficient antioxidant defense [47,69,70]. Additionally, while most data highlight the harmful effects of anesthetic exposure, some studies have documented context-dependent neuroprotection: for example, pre-conditioning and postconditioning with sevoflurane [79,95,96] or isoflurane [113,118] reduced neuroinflammatory responses and neuronal apoptosis in ischemia–reperfusion models. These variations in results may be attributed to the variation in the cell and animal models, the age of development of the animals, the anesthetic protocols (dose, frequency, and duration), and the type of injury model. Based on the above discussions of the current research, it is evident that further studies using clinically applicable models and methods need to be carried out to establish the significance of such molecular alterations and their implications in developing therapeutic strategies to alleviate the neurotoxicity induced by inhalational anesthesia.

## 8. Conclusions and Limitations

In conclusion, this review highlights an overview of the complex molecular mechanisms underlying the neurotoxicity of inhalation anesthetics in cellular and animal experimental models, with a focus on sevoflurane and isoflurane. In summary, these anesthetics induce significant alterations in gene expression (e.g., Bax/Bcl-2, miRNAs, lncRNAs, and DNA methylation), leading to modulation of signaling pathways (e.g., Nrf2/ARE, NF-κB, PI3K/Akt, and BDNF/TrkB) and activation of cellular responses including apoptosis, oxidative stress, ferroptosis, and neuroinflammation, which contribute to neuronal/glial injuries and potential long-term cognitive, sensory, and motor impairments, especially in the developing and aging brain of animals.

Animal models have given important mechanistic insights and promising neuroprotective strategies targeting these molecular alterations affected by sevoflurane and isoflurane. However, there are still several limitations and challenges in translating these findings to the clinical setting. Differences in brain maturation and anesthetic sensitivity between these species constitute challenges, especially with regard to safety and efficacy in humans. Particularly, many studies use exposure protocols or concentrations of inhalational anesthetics, which are not representative of standard clinical practice, and species differences limit direct extrapolation to humans. In addition, it is important to underline the key limitations in interpreting the available literature, including variability in methods that may impair the quality of individual studies, reporting bias, limited sample sizes, and lack of independent replication of findings. Furthermore, many studies do not perform neurobehavioral follow-up in the long term, which is another issue in assessing the long-term functional consequences of inhalational anesthetics. Therefore, rigorous methods and transparent reporting are necessary in the future to improve our understanding of the neurotoxicity of inhalational anesthetics.

## 9. Future Directions

Based on the presented findings in this review and in the context of clinical translation, future research should focus on the development of more rigorous and standardized preclinical models and long-term functional assessments to better evaluate the risk of neurotoxicity from inhalational anesthetics. Also, preclinical studies should be more focused on clarifying dose–response relationships, identifying susceptible populations, and standardizing anesthetic doses and exposure times to enable clinical translation. Furthermore, it seems valuable to identify reliable markers and risk factors for evaluating anesthesia-induced neurotoxicity to improve the safety of anesthesia in clinical practice, as well as to develop effective strategies to prevent or mitigate this serious adverse effect of inhalational anesthesia.

## Figures and Tables

**Table 1:** Gene alterations associated with sevoflurane-induced neurotoxicity in vitro experiments.

Reference	Specific Model	Anesthetic Protocol	Genes Alterations	Neurotoxic Event
***Human cell cultures***				
[59]	HN-h	5–15% for 6 h	hsa-miR-302e ↑ OXR1 ↓	Viability ↓ Apoptosis ↑
[60]	HCN-2	4% for 6 h	NKILA ↑ miR-205-5p ↓ ELAVL1 ↑	Viability ↓ Apoptosis ↑ Inflammation ↑ Oxidative stress ↑
[61]	M17	3% for 6 h	Sestrin-2 ↑ p53 ↑ Bax ↑ Bcl-2 ↓ cleaved caspase-3 ↑	Oxidative stress ↑ Mitochondrial dysfunction ↑ Apoptosis ↑
[39]	M17	2% for 36 h	DJ-1 ↑	Oxidative stress ↑ Apoptosis ↑
[62]	H4	4.1% for 6 h	Nrf2 ↑	Apoptosis ↑ Inflammation ↑ Oxidative stress ↑
[54]	H4	4.1% for 6 h	HSP70 ↑	Apoptosis ↑ Inflammation ↑ Oxidative stress ↑
[63]	U251	1.2%, 2.4%, and 3.6% for 3, 6, 12 h	SLC7A11 ↓ Ferroptosis regulatory factors ↓	Viability ↓ Ferroptosis ↑ Apoptosis ↑
[64]	U251	6% for 8 h	miR-211-5p ↑ SIRT1/PI3K/AKT ↓	Apoptosis ↑ Autophagy ↑
[65]	SH-SY5Y	4.1% for 6 h	m6A ↓ METTL14 ↓ DUSP6 ↓	Viability ↓ Apoptosis ↑
[66]	SH-SY5Y	3.4% for 2, 4, 6 h	Beclin-1 (2 h, 4 h ↑, 6 h ↓) Atg5 (2 h, 4 h ↑, 6 h ↓) LC3-II (2 h, 4 h ↑, 6 h ↓) BACE-1 (2 h, 4 h ↓, 6 h ↑) Aβ (2 h, 4 h ↓, 6 h ↑)	Apoptosis ↑ Autophagy (2 h, 4 h ↑, 6 h ↓)
***Animal cell cultures***				
[67]	Primary hippocampal neurons (rat)	3% for 12 h	Bax ↑ caspase-3 ↑ Bcl-2 ↓ p-p38 ↓ p-JNK ↓	Viability ↓ Apoptosis ↑
[68]	Primary hippocampal neurons (rat)	2% for 6 h	miR-128-3p ↓ NOVA-1 ↑	Viability ↓ Apoptosis ↑
[69]	Primary hippocampal neurons (rat)	4.1% for 6 h	NEAT1 ↑ Nrf2 ↓ HO-1 ↓	Apoptosis ↑ Oxidative stress ↑
[70]	Primary hippocampal neurons (rat)	3% for 3 h	Nrf2 ↑ HO-1 ↓ NQO1 ↓ GCL ↓ Prx1 ↓	Viability ↓ Apoptosis ↑ Oxidative stress ↑
[71]	Primary cortical neurons (rat)	4% for 6 h	EPOR ↑	Viability ↓ Apoptosis ↑ Oxidative stress ↑
[72]	Primary hippocampal neurons (rat)	3% for 12 h	SIRT1 ↑ mTOR ↓ Bax ↑ cleaved caspase-3 ↑ p62 ↓ LC3 ↑	Apoptosis ↑ Autophagy ↑
[73]	Primary hippocampal neurons (rat)	2%, 4%, 8% for 6, 12, 24 h	ATM/p53 ↑, JNK/p38 MAPK ↑, DNA stand breaks ↑, γ-H2AX ↑, Genes related to intracellular iron homeostasis ↑, Genes related to lipid peroxidation ↑	Viability ↓ Ferroptosis ↑
[74]	Primary hippocampal neurons (mice)	4.1% for 4 h	ApoE ↑	Viability ↓ Inflammation ↑
[75]	Primary hippocampal neurons (mice)	2.2% for 6 h	lncRNK Rian ↓	Viability ↓ Apoptosis ↑
[76]	BV2 Mice neurons and astrocytes	1% and 2% for 4 h	IL-6 ↑ (only in BV2, and astrocytes)	Inflammation ↑
[77]	BV2	1 mM	PCSK9 ↑, mtDNA ↑	Apoptosis ↑
[51]	BV2	3% for 6 h	C1q ↑ C3 ↑	Phagocytosis ↑
[78]	PC12	8% for 15 min	miR-424 ↓	Apoptosis ↑ Inflammation ↑

An upward arrow denotes increased values for the estimated parameter; a downward arrow denotes decreased values for the estimated parameter.

**Table 2:** Gene alterations associated with sevoflurane-induced neurotoxicity in neurodevelopmental animal models.

Reference	Specific Model	Anesthetic Protocol	Genes Alterations	Neurotoxic Event
***Prenatal exposure models***				
[80]	Neonatal mice (maternal exposure, C57BL/6J)	2.5% for 6 h	CXCL12 ↓ CXCR4 ↓	Epilepsy susceptibility ↑ Anxiety- and depression-like behaviors ↑ Interneuron migration ↓
[76]	Fetal mice (maternal exposure, C57BL/6J)	2% for 3 h	IL-6 ↑ IL-17 (n.c.) TNF-α (n.c.) IL-1β (n.c.)	Cognitive impairment ↑
[81]	Neonatal mice (maternal exposure, C57BL/6J)	3% for 2 h for 3 days	CD206 ↓ CD86 ↑ SOCS3 ↑	Neuroinflammation ↑ Cognitive impairment ↑
[63]	Neonatal mice (maternal exposure, C57BL/6J)	2.5% for 2 h	SLC7A11 ↓	Anxiety- like behaviors ↑ Cognitive impairment ↑ Hippocampal Damage ↑ Neuroinflammation ↑
***Neonatal exposure models***				
[82]	Neonatal mice (C57BL/6J)	3% for 6 h	BDNF ↓ TrkB ↓	Apoptosis ↑ Cognitive impairment ↑
[83]	Neonatal mice (C57BL/6J)	2.3% for 6 h	lncRNA (ENSMUST00000136025) ↑ Bcl2l11 (Bim) ↑	Apoptosis ↑
[84]	Neonatal rats (SD)	2.5% for 6 h	DISC1 ↓	Apoptosis ↑ Cognitive impairment ↑
[41]	Neonatal rats (SD)	1%, 2%, 4% for 1 h	miR-96 ↑ IGF1R ↓ Bax ↑ Caspase-3 ↑ Bcl-2 ↓	Apoptosis ↑ Cognitive impairment ↑
[85]	Neonatal mice	3% for 6 h	USP30 ↑	Neuroinflammation ↑ Cognitive impairment ↑
[86]	Neonatal mice (C57BL/6J)	2% for 6 h	MEG3 ↓ NLRP3 ↑ Caspase-1 ↑ IL-1β ↑ IL-18 ↑	Apoptosis ↑ Cognitive impairment ↑
[87]	Neonatal mice (C57BL/6J)	2.2% for 2 h for 3 days	miR-27a-3p ↑ PPARγ ↓ NOX1/4 ↑	Apoptosis ↑ Neuroinflammation ↑ Oxidative stress ↑ Cognitive impairment ↑
[75]	Neonatal mice (C57BL/6J)	2.2% for 2 h for 3 days	lncRNA Rian ↑	Apoptosis ↑ Cognitive impairment ↑
[88]	Neonatal mice (C57BL/6J)	3% for 2 h for 3 days	Rik-203 ↓	Neural differentiation ↓
[89]	Neonatal mice (C57BL/6J)	3% for 2 h for 3d	ERMN ↓, TYMS ↓	Cognitive impairment ↑ Myelination ↓ Disrupted folate metabolism
[90]	Neonatal mice (C57BL/6)	3% for 2 h for 3 d	VLCFAs/LCFAs ↓ DEGs ↓ (lipid metabolism) MBP ↓	Myelination ↓ Cognitive impairment ↑
[91]	Neonatal rats (SD)	3% for 2 h then 2.4% for 4 h	DNA methylation ↓ Arc ↑ JunB ↑	-
***Non-human primate models***				
[89]	Neonatal Rhesus Macaques	2.5–3% for 4 h for 3 d	ERMN ↓ TYMS ↓	Disrupted folate metabolism
[92]	Infant rhesus monkeys	2.5% for 9 h	Altered genes of lipid metabolism	Neuroinflammation ↑ Oxidative stress ↑

An upward arrow denotes increased values for the estimated parameter; a downward arrow denotes decreased values for the estimated parameter; n.c. denotes no significant change.

**Table 3:** Gene alterations associated with sevoflurane-induced neurotoxicity in adult animals.

Reference	Specific Model	Anesthetic Protocol	Genes Alterations	Neurotoxic Event
***Adult models***				
[78]	Rats (SD)	2% for 15 min	miR-424 ↓	Apoptosis ↑ Neuroinflammation ↑
[93]	Rats (SD)	2.5% for 6 h	miR-410-3p ↓ CXCR5 ↑	Apoptosis ↑ Neuroinflammation ↑ Cognitive impairment ↑
[94]	Mice (C57BL/6J)	4% for 5 h	lncRNA Gm5106 ↑ Hoxa5 ↑ miR-27-3p ↓ IL-6 ↑ IL-10 ↑ TNF-α ↑	Apoptosis ↑ Neuroinflammation ↑
[65]	Mice (C57BL/6J)	4% for 6 h	m6A ↓ DUSP6 ↓ METTL14 ↓	Cognitive impairment ↑
***Aged models***				
[97]	Aged rats (SD)	3% and 7% for 3 h	BDNF ↓	Apoptosis ↑ Oxidative stress ↑ Cognitive impairment ↑
[62]	Aged rats (SD)	2% for 45 min	Nrf2 ↑	Apoptosis ↑ Oxidative Stress ↑
[69]	Aged rats (SD)	3% for 5 h	NEAT1 ↑ Nrf2 ↓	Apoptosis ↑ Cognitive impairment ↑ Oxidative stress ↑
[98]	Aged rats (SD)	2.5% for 4 h	lncRNAs (Sancr 1, 2, and 3) ↑ Hif3a ↑ Prkcd ↑ Nfe2l2 ↑	Mitochondrial dysfunction ↑ Oxidative stress ↑
[54]	Aged rats (SD)	2% for 5 h	HSP70 ↑	Apoptosis ↑ Oxidative stress ↑ Neuroinflammation ↑
[99]	Aged rats (SD)	1.5% or 2.5% for 2 h (single or 5 d)	NF-κB ↑	Apoptosis ↑ Cognitive impairment ↑
[100]	Aged mice (C57BL/6J)	3% for 5 h	Kv1.3 ↑	Apoptosis ↑ Cognitive impairment ↑ Neuroinflammation ↑
[101]	Aged mice (C57BL/6J)	3% for 6 h	FKBP5 ↑	Cognitive impairment ↑ Neuroinflammation ↑
	Aged marmosets	1.5–2% for 6 h	FKBP5 ↑	-

An upward arrow denotes increased values for the estimated parameter; a downward arrow denotes decreased values for the estimated parameter.

**Table 4:** Gene alterations associated with isoflurane-induced neurotoxicity in vitro experiments.

Reference	Specific Model	Anesthetic Protocol	Genes Alterations	Neurotoxic Outcomes
***Human cell cultures***				
[102]	H4	2% for 6 h	Bax ↑ Bcl-2 ↑	Oxidative stress ↑ Apoptosis ↑
[103]	U251	2% for 6 h	STAT3 ↑ (mRNA) STAT3 ↓ (protein)	Oxidative stress ↑ Apoptosis ↑
[45]	SH-SY5Y	3% for 36 h	Nrf2/ARE ↑	Viability ↓ Oxidative stress ↑ Apoptosis ↑
[40]	SH-SY5Y	1%, 2%, and 3% for 36 h	DJ-1 ↑	Oxidative stress ↑ Apoptosis ↑
[104]	SH-SY5Y	3% for 24 h or 48 h	miR-214 ↓	Viability ↓ Oxidative stress ↑ Apoptosis ↑
***Animal cell cultures***				
[105]	Primary cortical neurons (mice)	2% for 6 h	DNA damage ↑	Apoptosis ↑ Oxidative stress ↑
[106]	Primary cortical neurons (mice)	1.5% for 4 h	LanCL1 ↓	Viability ↓ Synaptic impairment ↑ Oxidative stress ↑
[107]	Primary hippocampal neurons (rat)	1.5% for 6 h	miR-302b-3p ↑ PTEN ↓	Apoptosis ↑ Oxidative stress ↑
[43]	Primary hippocampal neurons (mice)	1.5% for 4 h	CDK5 ↓ p35 ↓ p25 ↑	Apoptosis ↑
[108]	HT22	3% for 48 h	miR-214 ↓	Viability ↓ Oxidative stress ↑ Apoptosis ↑
[76]	BV2 mice neurons and astrocytes	1% for 3 h	IL-6 ↑ (only in BV2, and astrocytes)	Inflammation ↑
[109]	BV2	2% for 24 h	TNF-α ↑ IL-1β ↑ COX-2 ↑ PGE2 ↑ Iba1 ↑ TREM2 ↓	Viability ↓ Oxidative stress ↑ Inflammation ↑
[110]	BV2	0.2, 0.4, 0.8 and 1.6% for 3, 6, 9 and 12 h	IL-1β ↑ TNF-α ↑ IL-6 ↑ IL-8 ↑ CD16 ↑ iNOS ↑	Inflammation ↑ Apoptosis ↑
[47]	PC12	2% for 6 h	miRNA-153 ↓	Oxidative stress ↑ Apoptosis ↑
[111]	PC12	2% for 12 h	miR-409 ↓	Oxidative stress ↑ Inflammation ↑ Apoptosis ↑

An upward arrow denotes increased values for the estimated parameter; a downward arrow denotes decreased values for the estimated parameter.

**Table 5:** Gene alterations associated with isoflurane-induced neurotoxicity in neurodevelopmental rodent models.

Reference	Specific Model	Anesthetic Protocol	Genes Alterations	Neurotoxic Event
***Prenatal exposure models***				
[76]	Fetal mice (maternal exposure, C57BL/6)	1% for 3 h	IL-6 ↑	Inflammation ↑
***Neonatal exposure models***				
[114]	Neonatal rats (SD)	1.3% for 3 h for 3 d	Bcl-2 ↓	Apoptosis ↑
[43]	Neonatal rats (SD)	1.5% for 4 h	CDK5 ↓ p35 ↓ p25 ↑	Apoptosis ↑ Cognitive impairment ↑
[103]	Neonatal mice (C57/BL6)	1.5% for 6 h	STAT3 (n.c.) (mRNA) STAT3 ↓ (protein)	Apoptosis ↑ Calcineurin activity ↑
[115]	Neonatal rats (SD)	1.5% for 6 h	miR-124 ↓ EGR1 ↑ BDNF ↓	Apoptosis ↑ Cognitive impairment ↑
[107]	Neonatal rats (SD)	1.5% for 6 h	miR-302b-3p ↑ PTEN ↓	Apoptosis ↑ Cognitive impairment ↑
[116]	Neonatal rats (SD)	0.75% for 6 h	BDNF ↓ TrkB ↓ TLR-4 ↑	Apoptosis ↑ Inflammation ↑ Cognitive impairment ↑
[110]	Neonatal rats (SD)	0.75% for 6 h	IL-1β ↑ TNF-α ↑ IL-6 ↑ IL-8 ↑ CD16 ↑ iNOS ↑ CD206 ↑ Arg1 ↑	Apoptosis ↑ Inflammation ↑
[106]	Neonatal mice (ICR)	1.5% for 4 h	LanCL1 ↓	Synaptic impairment ↑ Oxidative stress ↑ Cognitive impairment ↑

An upward arrow denotes increased values for the estimated parameter; a downward arrow denotes decreased values for the estimated parameter; n.c. denotes no significant change.

## Data Availability

No new data were created or analyzed in this study. Data sharing is not applicable to this article.

## Abbreviations

The following abbreviations are used in this manuscript:

TNF-α	Tumor Necrosis Factor-α
IL-1β	Interleukin-1β
IL-6	Interleukin-6
NF-κB	Nuclear Factor Kappa B
TIVA	Total Intravenous Anesthesia
GABA	γ-Aminobutyric Acid
NMDA	N-methyl-D-aspartate
POCD	Postoperative Cognitive Dysfunction
CDK5	Cyclin-Dependent Kinase 5
ROS	Reactive Oxygen Species
GLT1	Glutamate Transporter 1
HMGB1	High Mobility Group Box 1
miRNA	MicroRNA
lncRNA	Long Non-Coding RNA
HN-h	Human Hippocampal Cells
HCN-2	Human Cortical Neuronal Cells
M17	Human Neuroblastoma Cells
H4	Human Neuroglioma Cells
HT22	Mouse Hippocampal Neuronal Cells
U251	Human Glioblastoma Multiforme Cells
SH-SY5Y	Human Neuroblastoma Cells
PC12	Rat Pheochromocytoma Cells
BV2	Murine Microglial Cells
SD	Sprague Dawley
MDA	Malondialdehyde
MPO	Myeloperoxidase
SOD	Superoxide Dismutase
GSH	Glutathione
CAT	Catalase
CXCR5	C-X-C Motif Chemokine Receptor 5
NOX2	NADPH Oxidase 2
BDNF	Brain-Derived Neurotrophic Factor
mPTP	Mitochondrial Permeability Transition Pore
LDH	Lactate Dehydrogenase
COX2	Cyclooxygenase-2
PGE2	Prostaglandin E2
TREM2	Triggering Receptor Expressed on Myeloid Cells 2
iNOS	Inducible Nitric Oxide Synthase
Arg1	Arginase 1
GSK-3β	Glycogen Synthase Kinase-3β

## References

[1] Schiff J.H., Wagner S. (2016). Anesthesia Related Mortality? A National and International Overview. Trends Anaesth. Crit. Care.

[2] Poeran J., Mazumdar M., Memtsoudis S.G. (2014). Anesthesia, Outcomes, and Public Health: Changing Health Care While “Asleep”. Reg. Anesth. Pain Med..

[3] Dey J. (2025). Understanding Anesthesia Drugs: Mechanisms, Classifications, and Clinical Significance. ResearchGate.

[4] Franks N.P. (2008). General Anaesthesia: From Molecular Targets to Neuronal Pathways of Sleep and Arousal. Nat. Rev. Neurosci..

[5] Brown E.N., Lydic R., Schiff N.D. (2010). General Anesthesia, Sleep, and Coma. N. Engl. J. Med..

[6] Hemmings H.C., Riegelhaupt P.M., Kelz M.B., Solt K., Eckenhoff R.G., Orser B.A., Goldstein P.A. (2019). Towards a Comprehensive Understanding of Anesthetic Mechanisms of Action: A Decade of Discovery. Trends Pharmacol. Sci..

[7] Brown E.N., Pavone K.J., Naranjo M. (2018). Multimodal General Anesthesia: Theory and Practice. Anesth. Analg..

[8] Eger E.I. (2004). Characteristics of Anesthetic Agents Used for Induction and Maintenance of General Anesthesia. Am. J. Health-Syst. Pharm..

[9] Morgan M. (1983). Total Intravenous Anaesthesia. Anaesthesia.

[10] Oh T.K., Song I.-A., Jeon Y.-T. (2024). Comparison of Postoperative Outcomes after Cranial Neurosurgery Using Propofol-Based Total Intravenous Anesthesia versus Inhalation Anesthesia: A Nationwide Cohort Study in South Korea. Korean J. Anesthesiol..

[11] Folino T.B., Mahboobi S.K. (2026). Regional Anesthetic Blocks. StatPearls.

[12] Garmon E.H., Hendrix J.M., Huecker M.R. (2026). Topical, Local, and Regional Anesthesia and Anesthetics. StatPearls.

[13] Alshebly A.A., Alhwaiti A.S., Bahamdan O. (2025). Comparing Regional Anesthesia and General Anesthesia for Postoperative Pain Control in Abdominal Surgeries: A Systematic Review and Meta-Analysis. Cureus.

[14] Becker D.E., Reed K.L. (2012). Local Anesthetics: Review of Pharmacological Considerations. Anesth. Prog..

[15] Taylor A., McLeod G. (2020). Basic Pharmacology of Local Anaesthetics. BJA Educ..

[16] Jung J., Kim D.H., Son J., Lee S.K., Son B.S. (2019). Comparative Study between Local Anesthesia and General Anesthesia in the Treatment of Primary Spontaneous Pneumothorax. Ann. Transl. Med..

[17] Benzoni T., Agarwal A., Cascella M. (2026). Procedural Sedation. StatPearls.

[18] Godwin S.A., Burton J.H., Gerardo C.J., Hatten B.W., Mace S.E., Silvers S.M., Fesmire F.M. (2014). Clinical Policy: Procedural Sedation and Analgesia in the Emergency Department. Ann. Emerg. Med..

[19] Miller A.L., Theodore D., Widrich J. (2026). Inhalational Anesthetic. StatPearls.

[20] Gyorfi M.J., Kim P.Y. (2026). Halothane Toxicity. StatPearls.

[21] Sleigh J.W., Vizuete J.A., Voss L., Steyn-Ross A., Steyn-Ross M., Marcuccilli C.J., Hudetz A.G. (2009). The Electrocortical Effects of Enflurane: Experiment and Theory. Anesth. Analg..

[22] Yasuda N., Lockhart S.H., Eger E.I.I., Weiskopf R.B., Liu J., Laster M., Taheri S., Peterson N.A. (1991). Comparison of Kinetics of Sevoflurane and Isoflurane in Humans. Anesth. Analg..

[23] Vutskits L., Xie Z. (2016). Lasting Impact of General Anaesthesia on the Brain: Mechanisms and Relevance. Nat. Rev. Neurosci..

[24] Knuf K., Maani C.V. (2026). Nitrous Oxide. StatPearls.

[25] Becker D.E., Rosenberg M. (2008). Nitrous Oxide and the Inhalation Anesthetics. Anesth. Prog..

[26] Patel S.S., Goa K.L. (1996). Sevoflurane. Drugs.

[27] Lerman J., Davis P.J., Welborn L.G., Orr R.J., Rabb M., Carpenter R., Motoyama E., Hannallah R., Haberkern C.M. (1996). Induction, Recovery, and Safety Characteristics of Sevoflurane in Children Undergoing Ambulatory Surgery: A Comparison with Halothane. Anesthesiology.

[28] Kharasch E.D. (1995). Biotransformation of Sevoflurane. Anesth. Analg..

[29] Ludders J.W. (1992). Advantages and Guidelines for Using Isoflurane. Vet. Clin. N. Am. Small Anim. Pract..

[30] Khan J., Patel P., Liu M. (2026). Desflurane. StatPearls.

[31] Gouthami M., Sahaja G., Vivekanand (2025). A Comparative Study of Desflurane versus Sevoflurane on Return of Airway Reflexes after Discontinuation of Anaesthetic Agent. Eur. J. Cardiovasc. Med..

[32] TerRiet M.F., DeSouza G.J., Jacobs J.S., Young D., Lewis M.C., Herrington C., Gold M.I. (2000). Which Is Most Pungent: Isoflurane, Sevoflurane or Desflurane?. Br. J. Anaesth..

[33] Safavynia S.A., Goldstein P.A. (2018). The Role of Neuroinflammation in Postoperative Cognitive Dysfunction: Moving From Hypothesis to Treatment. Front. Psychiatry.

[34] Smith G.T., Chen T.J., Shah N.M., Agrest B., Grotticelli J. (2024). Anesthesia-Mediated Neuroinflammatory Sequelae in Post Operative Cognitive Dysfunction: Mechanisms and Therapeutic Implications. Front. Anesthesiol..

[35] Zhang M., Yin Y. (2023). Dual Roles of Anesthetics in Postoperative Cognitive Dysfunction: Regulation of Microglial Activation through Inflammatory Signaling Pathways. Front. Immunol..

[36] Qiao Y., Feng H., Zhao T., Yan H., Zhang H., Zhao X. (2015). Postoperative Cognitive Dysfunction after Inhalational Anesthesia in Elderly Patients Undergoing Major Surgery: The Influence of Anesthetic Technique, Cerebral Injury and Systemic Inflammation. BMC Anesthesiol..

[37] Pang Q.-Y., Duan L.-P., Jiang Y., Liu H.-L. (2021). Effects of Inhalation and Propofol Anaesthesia on Postoperative Cognitive Dysfunction in Elderly Noncardiac Surgical Patients: A Systematic Review and Meta-Analysis. Medicine.

[38] Hogarth K., Tarazi D., Maynes J.T. (2023). The Effects of General Anesthetics on Mitochondrial Structure and Function in the Developing Brain. Front. Neurol..

[39] Zhang Y., Li Y., Han X., Dong X., Yan X., Xing Q. (2018). Elevated Expression of DJ-1 (Encoded by the Human PARK7 Gene) Protects Neuronal Cells from Sevoflurane-Induced Neurotoxicity. Cell Stress Chaperones.

[40] Liu W., Guo Q., Hu X., Peng L., Zhou B. (2015). Induction of DJ-1 Protects Neuronal Cells from Isoflurane Induced Neurotoxicity. Metab. Brain Dis..

[41] Xu C., Niu J.-J., Zhou J.-F., Wei Y.-S. (2019). MicroRNA-96 Is Responsible for Sevoflurane-Induced Cognitive Dysfunction in Neonatal Rats via Inhibiting IGF1R. Brain Res. Bull..

[42] Qi Z., Tianbao Y., Yanan L., Xi X., Jinhua H., Qiujun W. (2017). Pre-Treatment with Nimodipine and 7.5% Hypertonic Saline Protects Aged Rats against Postoperative Cognitive Dysfunction via Inhibiting Hippocampal Neuronal Apoptosis. Behav. Brain Res..

[43] Wang W.-Y., Luo Y., Jia L.-J., Hu S.-F., Lou X.-K., Shen S.-L., Lu H., Zhang H.-H., Yang R., Wang H. (2014). Inhibition of Aberrant Cyclin-Dependent Kinase 5 Activity Attenuates Isoflurane Neurotoxicity in the Developing Brain. Neuropharmacology.

[44] Yang Y., Song S., Min H., Chen X., Gao Q. (2016). STAT3 Degradation Mediated by Calcineurin Involved in the Neurotoxicity of Isoflurane. NeuroReport.

[45] Wu Q., Shang Y., Shen T., Liu F., Zhang W. (2021). Biochanin A Protects SH-SY5Y Cells against Isoflurane-Induced Neurotoxicity by Suppressing Oxidative Stress and Apoptosis. Neurotoxicology.

[46] Ikonomidou C., Kaindl A.M. (2011). Neuronal Death and Oxidative Stress in the Developing Brain. Antioxid. Redox Signal..

[47] Shao D., Wu Z., Bai S., Fu G., Zou Z. (2019). The Function of miRNA-153 against Isoflurane-induced Neurotoxicity via Nrf2/ARE Cytoprotection. Mol. Med. Rep..

[48] Zhang Y., Xu Z., Wang H., Dong Y., Shi H.N., Culley D.J., Crosby G., Marcantonio E.R., Tanzi R.E., Xie Z. (2012). Anesthetics Isoflurane and Desflurane Differently Affect Mitochondrial Function, Learning, and Memory. Ann. Neurol..

[49] Cai J., Lin Y., Zhou B., Xiao F., Xu G., Lu J. (2024). SHARPIN Contributes to Sevoflurane-Induced Neonatal Neurotoxicity through up-Regulating HMGB1 to Repress M2 like-Macrophage Polarization. Metab. Brain Dis..

[50] Broad K.D., Hassell J., Fleiss B., Kawano G., Ezzati M., Rocha-Ferreira E., Hristova M., Bennett K., Fierens I., Burnett R. (2016). Isoflurane Exposure Induces Cell Death, Microglial Activation and Modifies the Expression of Genes Supporting Neurodevelopment and Cognitive Function in the Male Newborn Piglet Brain. PLoS ONE.

[51] Tian X., Xiong M., Jiang Z., Hong R., Wang H. (2025). Rutin Ameliorates Sevoflurane-Induced Neurotoxicity by Inhibiting Microglial Synaptic Phagocytosis through the Complement Pathway. Sci. Rep..

[52] Wang G., Liu H.-Y., Meng X.-W., Chen Y., Zhao W.-M., Li W.-T., Xu H.-B., Peng K., Ji F.-H. (2024). Complement C1q-Mediated Microglial Synaptic Elimination by Enhancing Desialylation Underlies Sevoflurane-Induced Developmental Neurotoxicity. Cell Biosci..

[53] Kong F., Zhang Y., Wang T., Zhong L., Feng C., Wu Y. (2023). Repeated Sevoflurane Exposures Inhibit Neurogenesis by Inducing the Upregulation of Glutamate Transporter 1 in Astrocytes. Eur. J. Neurosci..

[54] Liu M., Li M., Zhou Y., Zhou Q., Jiang Y. (2020). HSP90 Inhibitor 17AAG Attenuates Sevoflurane-Induced Neurotoxicity in Rats and Human Neuroglioma Cells via Induction of HSP70. J. Transl. Med..

[55] Bahmad H.F., Darwish B., Dargham K.B., Machmouchi R., Dargham B.B., Osman M., Khechen Z.A., El Housheimi N., Abou-Kheir W., Chamaa F. (2020). Role of MicroRNAs in Anesthesia-Induced Neurotoxicity in Animal Models and Neuronal Cultures: A Systematic Review. Neurotox. Res..

[56] Szymański M., Barciszewski J. (2002). Beyond the Proteome: Non-Coding Regulatory RNAs. Genome Biol..

[57] Niu Y., Yan J., Jiang H. (2022). Anesthesia and Developing Brain: What Have We Learned from Recent Studies. Front. Mol. Neurosci..

[58] Brodier E.A., Cibelli M. (2021). Postoperative Cognitive Dysfunction in Clinical Practice. BJA Educ..

[59] Yang L., Shen Q., Xia Y., Lei X., Peng J. (2018). Sevoflurane-induced Neurotoxicity Is Driven by OXR1 Post-transcriptional Downregulation Involving hsa-miR-302e. Mol. Med. Rep..

[60] Zhang Y., Chen C. (2023). Knockdown of lncRNA NKILA Suppresses Sevoflurane-Induced Neuronal Cell Injury Partially by Targeting miR-205-5p/ELAVL1 Axis. Gen. Physiol. Biophys..

[61] Yi W., Zhang Y., Guo Y., Li D., Li X. (2015). Elevation of Sestrin-2 Expression Attenuates Sevoflurane Induced Neurotoxicity. Metab. Brain Dis..

[62] Tian Y., Wu X., Guo S., Ma L., Huang W., Zhao X. (2017). Minocycline Attenuates Sevoflurane-Induced Cell Injury via Activation of Nrf2. Int. J. Mol. Med..

[63] Hu X., Zhang Y., Guo L., Xiao R., Yuan L., Liu F. (2024). Comprehensive Analysis of Bulk RNA-Seq and Single-Cell RNA-Seq Data Unveils Sevoflurane-Induced Neurotoxicity Through SLC7A11-Associated Ferroptosis. J. Cell. Mol. Med..

[64] Wang H., Cheng G., Zhang S., Qu H., Zhao X., Yang A., Sun X., Pan H. (2025). Sevoflurane: A Dual Modulator of miR-211-5p and Mitochondrial Apoptosis in Glioma Therapy. Mol. Med. Rep..

[65] Deng S., Mu G., Li J., Yu X., Li Q., Lu B. (2025). METTL14-Mediated m6A Modification of DUSP6 mRNA Participating in Postoperative Cognitive Dysfunction Due to Sevoflurane Anesthesia. J. Physiol. Sci. JPS.

[66] Xu L., Shen J., Dai S., Sun L., Chen X. (2020). Tetramethylpyrazine Attenuated Sevoflurane-Induced Neurotoxicity by Enhancing Autophagy through GPR50/CREB Pathway in SH-SY5Y Cells. Am. J. Chin. Med..

[67] Li S., He J. (2018). Pilose Antler Polypeptide Protects against Sevoflurane-mediated Neurocyte Injury. Mol. Med. Rep..

[68] Li D., Sun J., Yu M., Wang Y., Lu Y., Li B. (2022). The Protective Effects of miR-128-3p on Sevoflurane-Induced Progressive Neurotoxicity in Rats by Targeting NOVA1. J. Toxicol. Sci..

[69] Wang Y., Li N., Chen X., Zhao Y., Qu L., Cai D. (2024). Mechanistic Insights into Sevoflurane-Induced Hippocampal Neuronal Damage and Cognitive Dysfunction through the NEAT1/Nrf2 Signaling Axis in Aged Rats. Cell Biol. Toxicol..

[70] Zhou R., Li X., Li L., Zhang H. (2020). Theaflavins Alleviate Sevoflurane-Induced Neurocytotoxicity via Nrf2 Signaling Pathway. Int. J. Neurosci..

[71] Zhang D.-X., Zhang L.-M., Zhao X.-C., Sun W. (2017). Neuroprotective Effects of Erythropoietin against Sevoflurane-Induced Neuronal Apoptosis in Primary Rat Cortical Neurons Involving the EPOR-Erk1/2-Nrf2/Bach1 Signal Pathway. Biomed. Pharmacother..

[72] Li Y., Xiao C., Tan Y., Jing S. (2024). The Role of the SIRT1-mTOR Signaling Pathway in Regulating Autophagy in Sevoflurane-Induced Apoptosis of Fetal Rat Brain Neurons. Front. Biosci..

[73] Gu W., Pan T., Wang X., Kang L., Liu N., Piao M., Feng C. (2025). Sevoflurane Exposure Triggers Ferroptosis of Neuronal Cells Initiated by the Activation of ATM/P53 in the Neonatal Mouse Brain via JNK/P38 MAPK-Mediated Oxidative DNA Damage. Int. Immunopharmacol..

[74] Yang M., Lian N., Yu Y., Wang Y., Xie K., Yu Y. (2020). Coenzyme Q10 Alleviates Sevoflurane-induced Neuroinflammation by Regulating the Levels of Apolipoprotein E and Phosphorylated Tau Protein in Mouse Hippocampal Neurons. Mol. Med. Rep..

[75] Yu Y., Zhang W., Zhu D., Wang H., Shao H., Zhang Y. (2021). LncRNA Rian Ameliorates Sevoflurane Anesthesia-Induced Cognitive Dysfunction through Regulation of miR-143-3p/LIMK1 Axis. Hum. Cell.

[76] Hirotsu A., Iwata Y., Tatsumi K., Miyai Y., Matsuyama T., Tanaka T. (2019). Maternal Exposure to Volatile Anesthetics Induces IL-6 in Fetal Brains and Affects Neuronal Development. Eur. J. Pharmacol..

[77] Su T., Si Y. (2025). PCSK9 Exacerbates Sevoflurane-Induced Neuroinflammatory Response and Apoptosis by up-Regulating cGAS-STING Signal. Tissue Cell.

[78] Li Z., Wang T., Yu Y. (2022). miR-424 Inhibits Apoptosis and Inflammatory Responses Induced by Sevoflurane through TLR4/MyD88/NF-κB Pathway. BMC Anesthesiol..

[79] Chen C., Zuo J., Zhang H. (2022). Sevoflurane Post-Treatment Mitigates Oxygen-Glucose DeprivationinducedPyroptosis of Hippocampal Neurons by Regulating theMafb/DUSP14 Axis. Curr. Neurovasc. Res..

[80] Liang X., Jiang M., Xu H., Tang T., Shi X., Dong Y., Xiao L., Xie Y., Fang F., Cang J. (2023). Maternal Sevoflurane Exposure Increases the Epilepsy Susceptibility of Adolescent Offspring by Interrupting Interneuron Development. BMC Med..

[81] Tang X.L., Wang X., Fang G., Zhao Y.L., Yan J., Zhou Z., Sun R., Luo A.L., Li S.Y. (2021). Resveratrol Ameliorates Sevoflurane-Induced Cognitive Impairment by Activating the SIRT1/NF-κB Pathway in Neonatal Mice. J. Nutr. Biochem..

[82] Ding M.-L., Ma H., Man Y.-G., Lv H.-Y. (2017). Protective Effects of a Green Tea Polyphenol, Epigallocatechin-3-Gallate, against Sevoflurane-Induced Neuronal Apoptosis Involve Regulation of CREB/BDNF/TrkB and PI3K/Akt/mTOR Signalling Pathways in Neonatal Mice. Can. J. Physiol. Pharmacol..

[83] Chen X., Zhou X., Lu D., Yang X., Zhou Z., Chen X., Chen Y., He W., Feng X. (2016). Aberrantly Expressed Long Noncoding RNAs Are Involved in Sevoflurane-Induced Developing Hippocampal Neuronal Apoptosis: A Microarray Related Study. Metab. Brain Dis..

[84] Yang T.-T., Wei R., Jin F.-F., Yu W., Zhang F., Peng Y., Zhang S.-J., Qi S.-H., Liu J.-R. (2025). Dexmedetomidine Alleviates the Long-Term Neurodevelopmental Toxicity Induced by Sevoflurane in the Developing Brain. Dev. Neurosci..

[85] Gao T., Huang Z. (2024). Novel Insights into Sevoflurane-Induced Developmental Neurotoxicity Mechanisms. Epigenomics.

[86] Ma Z., Niu H., Qi H., Li Y. (2024). Functional Role of lncRNA MEG3 on Pyroptosis through Interacting with EZH2 and YTHDC1 in Postoperative Cognitive Dysfunction. Brain Res. Bull..

[87] Lv X., Yan J., Jiang J., Zhou X., Lu Y., Jiang H. (2017). MicroRNA-27a-3p Suppression of Peroxisome Proliferator-Activated Receptor-γ Contributes to Cognitive Impairments Resulting from Sevoflurane Treatment. J. Neurochem..

[88] Zhang L., Yan J., Liu Q., Xie Z., Jiang H. (2019). LncRNA Rik-203 Contributes to Anesthesia Neurotoxicity via microRNA-101a-3p and GSK-3β-Mediated Neural Differentiation. Sci. Rep..

[89] Zhang L., Xue Z., Liu Q., Liu Y., Xi S., Cheng Y., Li J., Yan J., Shen Y., Xiao C. (2019). Disrupted Folate Metabolism with Anesthesia Leads to Myelination Deficits Mediated by Epigenetic Regulation of ERMN. EBioMedicine.

[90] Jiang S., Cao T., Li J., Di L., Wang X., Xie Z., Huang L. (2025). Neonatal Sevoflurane Exposure Disrupted Fatty Acids Metabolism, Leading to Hypomyelination and Neurological Impairments. Biomed. Pharmacother..

[91] Chastain-Potts S.E., Tesic V., Tat Q.L., Cabrera O.H., Quillinan N., Jevtovic-Todorovic V. (2020). Sevoflurane Exposure Results in Sex-Specific Transgenerational Upregulation of Target IEGs in the Subiculum. Mol. Neurobiol..

[92] Liu F., Rainosek S.W., Frisch-Daiello J.L., Patterson T.A., Paule M.G., Slikker W.J., Wang C., Han X. (2015). Potential Adverse Effects of Prolonged Sevoflurane Exposure on Developing Monkey Brain: From Abnormal Lipid Metabolism to Neuronal Damage. Toxicol. Sci..

[93] Su R., Sun P., Zhang D., Xiao W., Feng C., Zhong L. (2019). Neuroprotective Effect of miR-410-3p against Sevoflurane Anesthesia-Induced Cognitive Dysfunction in Rats through PI3K/Akt Signaling Pathway via Targeting C-X-C Motif Chemokine Receptor 5. Genes Genom..

[94] Zhu Z., Ma L. (2021). Sevoflurane Induces Inflammation in Primary Hippocampal Neurons by Regulating Hoxa5/Gm5106/miR-27b-3p Positive Feedback Loop. Bioengineered.

[95] Shang S., Sun F., Zhu Y., Yu J., Yu L., Shao W., Wang Z., Yi X. (2023). Sevoflurane Preconditioning Improves Neuroinflammation in Cerebral Ischemia/Reperfusion Induced Rats through ROS-NLRP3 Pathway. Neurosci. Lett..

[96] Peng L., Yang M., Liu J., Yin T., Li J., Sun S., Zhu H., Wang S. (2026). Sevoflurane Ameliorates Cerebral Ischemia–Reperfusion Injury by Modulating Mitochondrial Dynamics and Attenuating Apoptosis via Shh-YAP1 Signaling Pathway. Mol. Brain.

[97] Xu Z., Qian B. (2020). Sevoflurane Anesthesia-Mediated Oxidative Stress and Cognitive Impairment in Hippocampal Neurons of Old Rats Can Be Ameliorated by Expression of Brain Derived Neurotrophic Factor. Neurosci. Lett..

[98] Qu Y., Li H., Shi C., Qian M., Yang N., Wang L., Gao X., Ni C. (2020). lncRNAs Are Involved in Sevoflurane Anesthesia-Related Brain Function Modulation through Affecting Mitochondrial Function and Aging Process. BioMed Res. Int..

[99] Guo S., Liu L., Wang C., Jiang Q., Dong Y., Tian Y. (2018). Repeated Exposure to Sevoflurane Impairs the Learning and Memory of Older Male Rats. Life Sci..

[100] Li B., Gao Y., Han H., Wang Z., Zhang Y., Yu L., Ling Y. (2025). Pharmacological Inhibition of Kv1.3 Channel Reduces Sevoflurane-Induced Cognitive Impairment through NLRP3-Dependent Microglial Modulation. Brain Res. Bull..

[101] Zhang L., Zhu J., Miao Z., Mao H., Jiang H. (2026). Mechanisms by Which Sevoflurane Affects Cognitive Function in Aged Marmosets and Mice: Up-Regulation of FKBP5 Expression in Brain Microglia. Med. Gas. Res..

[102] Zhang Y., Dong Y., Wu X., Lu Y., Xu Z., Knapp A., Yue Y., Xu T., Xie Z. (2010). The Mitochondrial Pathway of Anesthetic Isoflurane-Induced Apoptosis. J. Biol. Chem..

[103] Yang Y., Song S., Wang H., Ma Z., Gao Q. (2024). The Antioxidative Effect of STAT3 Involved in Cellular Vulnerability to Isoflurane. BMC Neurosci..

[104] Wu Q., Shang Y., Shen T., Liu F., Xu Y., Wang H. (2019). Neuroprotection of miR-214 against Isoflurane-Induced Neurotoxicity Involves the PTEN/PI3K/Akt Pathway in Human Neuroblastoma Cell Line SH-SY5Y. Arch. Biochem. Biophys..

[105] Bai T., Dong D.-S., Pei L. (2013). Resveratrol Mitigates Isoflurane-Induced Neuroapoptosis by Inhibiting the Activation of the Akt-Regulated Mitochondrial Apoptotic Signaling Pathway. Int. J. Mol. Med..

[106] Xie W., Xi Y., Dong D., Liu S., Ma Z., Peng L., Xia T., Gu X. (2024). LanCL1 Protects Developing Neurons from Long-Term Isoflurane Anesthesia-Induced Neurotoxicity. Exp. Neurol..

[107] Li L., Lu S., Fan X. (2021). Silencing of miR-302b-3p Alleviates Isoflurane-Induced Neuronal Injury by Regulating PTEN Expression and AKT Pathway. Brain Res. Bull..

[108] Zhang G., Sun C., Zhou G., Zhang Q. (2024). Luteolin Protects Mouse Hippocampal Neuronal Cells against Isoflurane-Induced Neurotoxicity through miR-214/PTEN/Akt Pathway. Neurotoxicology.

[109] Hu R., He Y., Chen Z. (2021). Maprotiline Ameliorates Isoflurane-Induced Microglial Activation via Regulating Triggering Receptor Expressed in Myeloid Cells 2 (TREM2). Bioengineered.

[110] Jiang T., Xu S., Shen Y., Xu Y., Li Y. (2021). Genistein Attenuates Isoflurane-Induced Neuroinflammation by Inhibiting TLR4-Mediated Microglial-Polarization in Vivo and in Vitro. J. Inflamm. Res..

[111] Qi Y., Chen L., Shan S., Nie Y., Wang Y. (2020). Vitexin Improves Neuron Apoptosis and Memory Impairment Induced by Isoflurane via Regulation of miR-409 Expression. Adv. Clin. Exp. Med..

[112] Klenke S., Specking C., Stegen M., Engler A., Peters J. (2020). Methylation in HT22 Cells and Primary Hippocampal Neurons with and without Isoflurane Exposure. BMC Anesthesiol..

[113] Cao L., Feng C., Li L., Zuo Z. (2012). Contribution of microRNA-203 to the Isoflurane Preconditioning-Induced Neuroprotection. Brain Res. Bull..

[114] Yong J., Yan L., Wang J., Xiao H., Zeng Q. (2018). Effects of Compound 21, a Non-peptide Angiotensin II Type 2 Receptor Agonist, on General Anesthesia-induced Cerebral Injury in Neonatal Rats. Mol. Med. Rep..

[115] Yang W., Guo Q., Li J., Wang X., Pan B., Wang Y., Wu L., Yan J., Cheng Z. (2019). microRNA-124 Attenuates Isoflurane-Induced Neurological Deficits in Neonatal Rats via Binding to EGR1. J. Cell. Physiol..

[116] Sun N., Chu L., Yuan L., Qi Z. (2019). Inactivation of P2YR12 Contributes to Isoflurane-Induced Neuronal Injury by Altering TLR-4/BDNF/TNF-α. Folia Neuropathol..

[117] Si J., Jin Y., Cui M., Yao Q., Li R., Li X. (2021). Neuroprotective Effect of miR-212-5p on Isoflurane-Induced Cognitive Dysfunction by Inhibiting Neuroinflammation. Toxicol. Mech. Methods.

[118] Li H., Yin J., Li L., Deng J., Feng C., Zuo Z. (2013). Isoflurane Postconditioning Reduces Ischemia-Induced Nuclear Factor-κB Activation and Interleukin 1β Production to Provide Neuroprotection in Rats and Mice. Neurobiol. Dis..

[119] Zhang X., Fan X., Li F., Qiu J., Zhang Y. (2020). Effects of PYRIN-Containing Apaf1-like Protein 1 on Isoflurane-Induced Postoperative Cognitive Dysfunction in Aged Rats. Mol. Med. Rep..

[120] Li S.-Y., Chen X., Chen Y.-L., Tan L., Zhao Y.-L., Wang J.-T., Xiang Q., Luo A.-L. (2013). Role of GSK-3β in Isoflurane-Induced Neuroinflammation and Cognitive Dysfunction in Aged Rats. J. Huazhong Univ. Sci. Technol. [Med. Sci.].

